# Effects of Light Intensity and Nitrogen Starvation on Glycerolipid, Glycerophospholipid, and Carotenoid Composition in *Dunaliella tertiolecta* Culture

**DOI:** 10.1371/journal.pone.0072415

**Published:** 2013-09-05

**Authors:** So-Hyun Kim, Kwang-Hyeon Liu, Seok-Young Lee, Seong-Joo Hong, Byung-Kwan Cho, Hookeun Lee, Choul-Gyun Lee, Hyung-Kyoon Choi

**Affiliations:** 1 College of Pharmacy, Chung-Ang University, Seoul, Republic of Korea; 2 College of Pharmacy, Kyungpook National University, Daegu, Republic of Korea; 3 Institute of Industrial Biotechnology, Department of Biological Engineering, Inha University, Incheon, Republic of Korea; 4 Department of Biological Sciences, KAIST, Daejeon, Republic of Korea; 5 College of Pharmacy, Gachon University, Incheon, Republic of Korea; RIKEN PSC, Japan

## Abstract

Time-course variation of lipid and carotenoid production under high light (300 μE/m^2^s) and nitrogen starvation conditions was determined in a *Dunaliella tertiolecta* strain. Nanoelectrospray (nanoESI) chip based direct infusion was used for lipid analysis and ultra-performance liquid chromatography (UPLC) coupled with a photodiode array (PDA) or atmospheric chemical ionization mass spectrometry (APCI-MS) was used for carotenoid analysis. A total of 29 lipids and 7 carotenoids were detected. Alterations to diacylglyceryltrimethylhomoserine (DGTS) and digalactosyldiacylglycerol (DGDG) species were significant observations under stress conditions. Their role in relation to the regulation of photosynthesis under stress condition is discussed in this study. The total carotenoid content was decreased under stress conditions, while ã-carotene was increased under nitrate-deficient cultivation. The highest productivity of carotenoid was attained under high light and nitrate sufficiency (HLNS) condition, which result from the highest level of biomass under HLNS. When stress was induced at stationary phase, the substantial changes to the lipid composition occurred, and the higher carotenoid content and productivity were exhibited. This is the first report to investigate the variation of lipids, including glycerolipid, glycerophospholipid, and carotenoid in *D. tertiolecta* in response to stress conditions using lipidomics tools.

## Introduction

*Dunaliella tertiolecta* was on the focus of this study because of its relatively high oil content, rapid growth rate, and high tolerance to extreme conditions such as high salinity [1]–[3]. It facilitates the extraction process because of its lack of a rigid cell wall, making it a promising resource for producing biofuel and pharmaceutical materials such as carotenoids [4]. Until now, most of studies on *D. tertiolecta* have only focused on analyzing content and composition of fatty acids and measuring cell growth or total lipid content [5], [6]. However, there were no reports of individual lipid species and carotenoid profiling in *D. tertiolecta* cultures. Moreover, only a few of reports of lipidomics studies based on mass spectrometry on other microalgae species have been published to date [7]–[9].

Although traditional analytical technologies, particularly thin-layer chromatography (TLC), have been used as the major tools to investigate lipids in microalgae [10], [11], after developing direct-infusion electrospray ionization-mass spectrometry (ESI-MS), it has been adopted by investigators to identify algal lipidome because of its relatively simple analytical process and potential for high-throughput analysis [12].

To survive in extreme environments, microalgae contain characteristic lipid species such as betaine lipids and alter the chain length and/or the degree of saturation. In other words, they induce specific alterations in lipids to resist against stress conditions [13]. However, lipidomics research regarding the pathways and alterations of individual lipid species in microalgae under various stress condition has been lacking until now, despite the importance of elucidating the biological role of lipids as a defense response to stress conditions.

The hypothesis of this study is that considerable alterations to lipids species and their compositions would occur under nitrogen-starvation or high light intensity conditions in *D. tertiolecta* cells. The objectives of this study are to identify individual lipid species and carotenoid, and investigate the change tendency of lipids over the stress time point and duration under various stress condition. The carotenoid content and productivity under several different stress conditions were also determined.

## Materials and Methods

### Chemicals and Reagents

HPLC-grade acetonitrile, methanol, chloroform, water and hexane were purchased from Honeywell Burdick & Jackson (Muskegon, MI, USA). HPLC-grade methyl tertiary butyl ether (MTBE), butylated hydroxyl toluene (BHT), β-apo-8′-carotenal, and ammonium acetate were purchased from Sigma-Aldrich (St. Louis, MO, USA). Carotenoid (neoxanthin, NEO; violaxanthin, VIO; anthraxanthin, ANT; zeaxanthin, ZEA; lutein, LUT; β-cryptoxanthin, βCRYP; lycopene, LYC; neurosporene, NEU; γ-carotene, γCAR; α-carotene, αCAR; β-carotene, βCAR;) standards were obtained from Carotenature (Lupsingen, Switzerland). LC-MS grade formic acid was purchased from Fisher Scientific (Loughborough, UK). Phospholipids (phosphatidylcholine, PC; phosphatidylglycerol, PG; phosphatidylinositol, PI; phosphatidylethanolamine, PE; lysophosphatidylcholine, LPC; lysophosphatidylinositol, LPI) and two glycolipids (monogalactosyldiacylglycerol, MGDG; digalactosyldiacylglycerol, DGDG) standards used in this study were purchased from Avanti Polar Lipids (Alabaster, AL, USA). A standard of sulfoquinovosyldiacylglycerol (SQDG) was obtained from Lipid Products (Redhill, UK). All chemicals were of analytical grade or higher purity.

### Strain Culture

The green microalga *D. tertiolecta* (UTEX LB999) was obtained from the Culture Collection of Algae at the University of Texas at Austin (UTEX). Cells were grown photoautotrophically in three folded f/2 medium [14] except vitamin solution at 21°C and irradiated with fluorescent lamps at 50 μE/m^2^s. The optimal light intensity was determined in a preliminary experiment, as described previously [15]. A constant nitrate concentration of 2.68 mM was maintained by supplying NO_3_ stock solution every 24 hours after measuring the NO_3_ concentration. *D. tertiolecta* cultures were set up in 400 mL bubble-column photobioreactors [16] at a 0.1 g/L fresh cell weight (FCW) inoculation concentration and a flow rate of 0.1 vvm bubbled air that contained 2% CO_2_.

### Stress Conditions

A stress was triggered in middle of the exponential phase and the beginning of the stationary phase. Based on a preliminary study, each point was determined by the fresh cell weight and cell concentration, which were 1.7 g/L (4.3×10^7^ cells/L) and 3.2 g/L (1.3×10^7^ cells/L), respectively. The cells were harvested at that point, and then resuspended into stress culture conditions. For nitrogen starvation and/or high light stress, cells were transferred to nitrate-free mediums and/or new cultures under the continuous illumination at 300 μE/m^2^s. Therefore, the following combinations of shifts in culture conditions of *D. tertiolecta* were investigated: high light intensity and nitrate deficiency (HLND), high light intensity and nitrate sufficiency (HLNS), low light intensity and nitrate deficiency (LLND), and low light intensity and nitrate sufficiency (LLNS); the LLNS condition was the same as the initial condition. All treatments were performed in duplicate and the cells were harvested at 0 (control), 12, 36, and 72 h after starting the stress at each phase. When cells were harvested, 400 mL of the cell suspensions were centrifuged at 1500 rpm (UNION 32R PLUS, Hanil Science Industrial. Co., Incheon, Republic of Korea) for 5 min and washed with distilled water (Milli-Q system, Millipore, Bedford, MA, USA). This process was repeated four times. Dry cell weights (DCW) were measured after freeze-drying, and cells were kept in labeled tubes at −80°C for storage.

### Lipid Extraction

Glycerolipids and glycerophospholipids, were extracted with a modified Folch method [17]. Three hundred microliters of methanol and 600 µL of chloroform were added to 1 mg of dried cells and vortexed. Then, the mixture was incubated for 1 h at room temperature in a shaker, and then phase separation was induced by adding 250 µL of water. The mixture was centrifuged at 1,000 *g* for 10 min and the lower phase was collected using a Pasteur pipet. Then the upper phase was extracted again as was done in the above procedure with 400 µL of the solvent mixture, whose composition was equivalent to the assumed composition of the lower phase (chloroform/methanol/water, 86∶14∶1, v/v/v) and then combined with the first lower phase. Five hundred microliters of the combined organic phase aliquot was taken and then dried with nitrogen gas. The dried extracts were stored at −80°C for subsequent analysis.

A method based on Bohoyo-Gil et al [18] and Scaife et al [19] were modified to extract carotenoids from *D. tertiolecta*. Initially, 10 mg of dried *D. tertiolecta* sample was extracted with 1 mL of acetone and vortexed for 20 sec. After a 5 min sonication, the tube was centrifuged at 1,000 *g* for 10 min at 4°C (Model 1730MR, Gyrozen Co., Ltd, Daejeon, Republic of Korea). The extraction was repeated twice until colorless. The supernatant was collected separately from each sample and the extracts were passed through syringe filters (1.7 cm^2^) with a 0.45-μm pore size polytetrafluoroethylene (PTFE) membrane (Minisart SRP 15, Sartorius, Germany). After filtration, 0.7 mL of each sample solution was transferred into vials and 0.7 mL of hexane with 0.1% BHT and 0.2 mL of water were added and the mixture was vigorously shaken. The 0.5 mL hexane layer (upper layer) was separated, and this extract was dried under a nitrogen gas flow and reconstituted in 0.5 mL of acetonitrile: methanol (70∶30, v/v). All extracts were protected from light and treated using amber glass vials with screw caps to avoid degradation of carotenoids.

### Analysis of Glycerolipids and Glycerophospholipids

Lipid analysis was performed using electrospray ionization-tandem mass spectrometry (ESI-MS/MS) on a linear ion-trap mass spectrometer (LTQ-XL, ThermoFisher Scientific, San Jose, CA, USA) equipped with an automated nanoinfusion/nanospray source (Triversa NanoMate System, Advion, Ithaca, NY, USA). The ionization voltage was set to 1.4 kV, gas pressure to 0.4 psi, and the source was controlled by Chipsoft 8.3.1 software (Advion, Ithaca, NY, USA). All lipid samples were dissolved in 500 µL of methanol: chloroform (9∶1, v/v) containing 7.5 mM ammonium acetate, and 5 μL of the reconstituted samples were infused. The infusion of the extracted lipid samples was performed using an Advion ESI chip with 5.5 μm ID emitter nozzles.

Fifteen microliters of each sample were randomly loaded into a 96-well plate (Eppendorf, Germany) to avoid bias in analysis. A standard mixture of glycerophospholipids (PC, PE, PI, and PG at concentrations of 250 µg/mL each) and a quality-control (QC) sample (pooled sample) were loaded at the beginning, middle, and end of the batch. The 96-well plate was sealed with aluminum film (ThermoFisher Scientific, San Jose, CA, USA) using a heat sealer and then placed on the NanoMate cooling plate, which was set to 5°C to prevent evaporation of the solvent. Full-scan spectra were collected at the 400–1200 *m/z* and the 500–1300 *m/z* ranges in positive and negative-ion mode, respectively. The capillary temperature was set to 200°C. The tube lens was set to 100 V. The mass spectra of each sample were acquired in profile mode over 2 min. A collision-induced dissociation (CID) was performed with over an isolated width of 3 *m/z* units, with 35% collision energy. The tandem mass spectrometry (MS/MS) triggering threshold was set to 500, with a default charge state of 1. Dynamic exclusion parameters were a 60 s repeat duration, 60 s exclusion duration, and a 50 exclusion list size.

### Analysis of Carotenoids

The ultra-performance liquid chromatography (UPLC) system was an Accela LC (ThermoFisher Scientific, San Jose, CA, USA) equipped with a degasser, Accela 600 pump, and Accela AS autosampler. The column was selected as a Zorbax Eclipse plus C18 column, 2.1 mm×100 mm and 1.8 µm particle size from Agilent Technologies (Böblingen, Germany) [18], and the column oven was set to a temperature of 25°C. The temperature of the auto-sampler tray was set to 4°C. The injection volume was 5 µL for each reconstituted sample and the elution flow rate was set to a constant 200 µL/min. The mobile phases were a 70∶20∶10 mixture of acetonitrile/methanol/MTBE (solvent A) and 0.1% formic acid (solvent B). The gradient program was as follows: 75% A for 4 min, followed by 98% A between 12 and 40 min. The equilibrium time was 5 min after each run, with 75% solvent A. The separation efficiency of UPLC was evaluated with the separation factor (α) and resolution (Rs).

The LC effluent was pumped through a Accela photodiode array (PDA) (ThermoFisher Scientific, San Jose, CA, USA) and LTQ-XL equipped with an atmospheric chemical ionization (APCI) source. It was reported that APCI is a more powerful technique for ionizing the carotenoids than electrospray ionization (ESI) or the atmospheric pressure photoionization (APPI) [20]. The flow rates of the nitrogen sheath gas and auxiliary gas were set to 25 and 5 (arbitrary units), respectively. The capillary temperature was 275°C and the temperature of the APCI vaporizer was set to 350°C. The voltages of the source, capillary, and tube lens were 6 kV, 41 V, and 100 V, respectively, in positive mode. Mass spectrometer was operated in full-scan mode from 500–700 *m/z*.

### Carotenoid Standards

One hundred micrograms per mililiter of standard stock solutions were prepared in hexane containing 0.1% BHT (w/v) in order to increase solution stability [18], [21]. Solutions were stored in amber vials at −80°C. Standard working solutions were prepared from the stock solution by the appropriate dilution in acetonitrile/methanol (70∶30, v/v). Standard mixtures were analyzed on three separate days to assess inter-day variability, which was performed by measuring the retention time (RT) and area ratio of carotenoids on the three different days, with three determinations each day.

### Data Acquisition and Statistical Analysis

MS/MS spectra were analyzed manually for the identification of lipid species using an in-house library, which was built using lipid standards and authentic references. The nominal ion mass spectra, which averaged the scans between 0.5–1.0 min, was extracted using Xcalibur software (ThermoFisher Scientific, San Jose, CA, USA). To normalize the spectrum, the average of the sum of intensities from the QC samples was divided by the sum of the intensities of each sample spectra, and then each value (fold) was multiplied by the intensity of each lipid species in that sample.

The normalized intensities of lipid species under stress conditions were compared to that under control conditions (LLNS). Significant differences in intensity levels of lipid species were determined by one-way analysis of variance using PASW Statistics 18 software (IBM, Somers, NY, USA), followed by the Tukey's significant-difference test. The level of statistical significance was set at *p*<0.05.

### Identification and Quantification of Carotenoids

The identification of various carotenoids was accomplished by a comparison of the retention times and the value of [M+H]^+^ with standards and sample peaks. The standard curves were obtained using a UPLC-PDA system (measuring at 460 nm) [18]. An internal standard, β-apo-8′-carotenal, was added to various concentrations of each carotenoids standards for quantification at a fixed concentration (2.5 µg/mL) [22]. Five concentrations, 0.3125, 0.625, 1.25, 2.5, and 5 µg/mL, were prepared for VIO and αCAR, 0.625, 1.25, 2.5, 5, and 10 µg/mL for γCAR and βCAR, 1.25, 2.5, 5, 10, and 20 µg/mL for NEO and ANT, and 3.125, 6.25, 12.5, 25, and 50 µg/mL for LUT. Next, all standard solutions were injected into the UPLC in triplicate. The standard calibration curves were obtained by plotting concentration against area ratio between components and the internal standard using Quanbrowser (Version 2.5.6, Thermo Electron Corporation, Hemel Hempstead, UK). The limit of detection (LOD) and the limit of quantitation (LOQ) were determined at signal-to-noise ratios of 3.3 and 10, respectively, as described in a previous report [23].

## Results and Discussion

### 1. Identification of Glycerolipids and Glycerophospholipids

**Figure 1:**
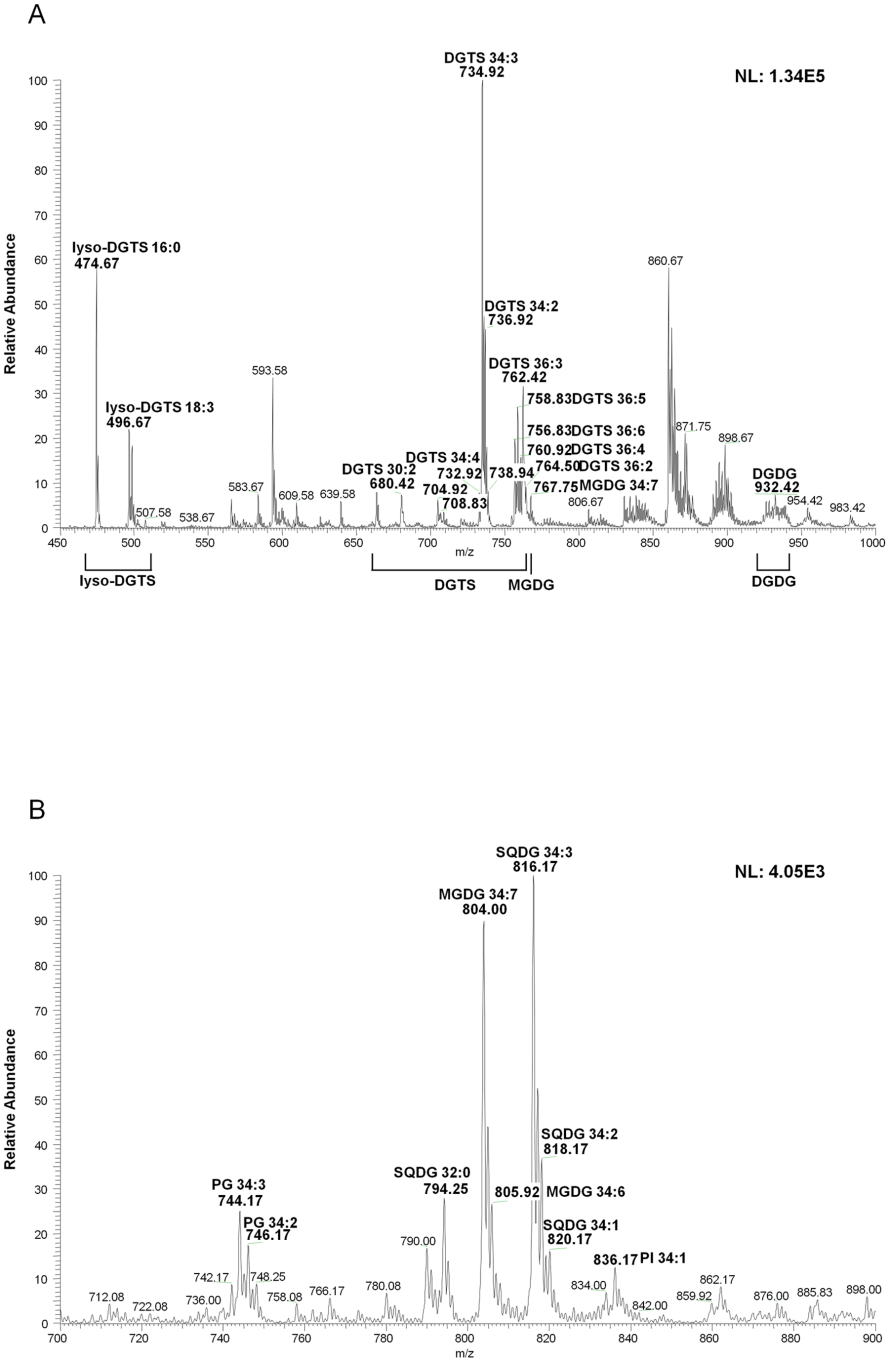
Analyses of glycerolipids and glycerophospholipids extracted from *D. tertiolecta* by direct infusion into electrospray ionization-tandem mass spectrometry (ESI-MS/MS) in both the (A) positive- and (B) negative-ion modes. DGTS, diacylglyceryltrimethylhomoserine; MGDG, monogalactosyldiacylglycerol; DGDG, digalactosyldiacylglycerol; SQDG, sulfoquinovosyldiacylglycerol; PG, phosphatidylglycerol; PI, phosphatidylinositol. *m/z*, mass-to-charge ratio. NL, normalized intensity level.

A representative pooled sample of *D. tertiolecta* was used for identifying lipids. In the positive mode, 12 of DGTS, 2 of lyso-DGTS, 1 of MGDG, and 6 of DGDG were detected (Fig. 1A, Table 1). DGTS is analyzed in positive ionization mode as [M + H]^+^, while MGDG and DGDG were detected as [M + Na]^+^. The specific diagnostic ions were used to identify each class of lipids. For example, DGTS species were identified by the fragment as an ion at m/z 236, corresponding to its polar head group of [C_10_H_22_NO_5_] (Fig. S1) [7].

**Table 1:** Identification of lipid species from *D. tertiolecta* by ESI-tandem mass spectrometry equipped with an automated nanoinfusion/nanospray source.

No.	Lipid molecular species^a^ (total acyl carbons: total double bonds)	Proposed composition	Ion mode	Ion species	Nominal mass used in lipid profiling	Theoret-ical exact mass (Da)	Element composition
1	DGTS 30∶2	C 16∶0/14∶2	(+)	[M + H]^+^	680	680.5465	C_40_H_74_O_7_N^+^
2	DGTS 32∶4	C 16∶0/16∶4	(+)	[M + H]^+^	705	704.5465	C_42_H_74_O_7_N^+^
3	DGTS 32∶2	C 16∶0/16∶2	(+)	[M + H]^+^	709	708.5778	C_42_H_78_O_7_N^+^
4	DGTS 34∶4	C 18∶4/16∶0	(+)	[M + H]^+^	733	732.5778	C_44_H_78_O_7_N^+^
5	DGTS 34∶3	C 18∶3/16∶0	(+)	[M + H]^+^	735	734.5935	C_44_H_80_O_7_N^+^
6	DGTS 34∶2	C 18∶2/16∶0	(+)	[M + H]^+^	737	736.6091	C_44_H_82_O_7_N^+^
7	DGTS 34∶1	C 18∶1/16∶0	(+)	[M + H]^+^	739	738.36248	C_44_H_84_O_7_N^+^
8	DGTS 36∶6	C 18∶3/18∶3	(+)	[M + H]^+^	757	756.5778	C_46_H_78_O_7_N^+^
9	DGTS 36∶5	C 18∶2/18∶3	(+)	[M + H]^+^	759	758.5935	C_46_H_80_O_7_N^+^
10	DGTS 36∶4	C 18∶2/18∶2	(+)	[M + H]^+^	761	760.6091	C_46_H_82_O_7_N^+^
11	DGTS 36∶3	C 18∶1/18∶2	(+)	[M + H]^+^	762	762.6248	C_46_H_84_O_7_N^+^
12	DGTS 36∶2	C 18∶1/18∶1, C18∶2/C18∶0	(+)	[M + H]^+^	765	764.6404	C_46_H_86_O_7_N^+^
13	LysoDGTS 16∶0	C 16∶0	(+)	[M + H]^+^	475	474.3795	C_26_H_52_O_6_N^+^
14	LysoDGTS 18∶3	C18∶3	(+)	[M + H]^+^	497	496.3638	C_28_H_50_O_6_N^+^
15	MGDG 34∶7	C 18∶3/16∶4	(+)	[M + Na]^+^	768	767.4710	C_43_H_68_O_10_Na^+^
			(−)	[M + OAc]^−^	804	803.4946	C_45_H_71_O_12_^−^
16	MGDG 34∶6	C 18∶3/16∶3	(−)	[M + OAc]^−^	806	805.5102	C_45_H_73_O_12_^−^
17	DGDG 34∶7	C 18∶3/16∶4	(+)	[M + Na]^+^	930	929.5238	C_49_H_78_O_15_Na^+^
18	DGDG 34∶6	C 18∶3/16∶3	(+)	[M + Na]^+^	932	931.5395	C_49_H_80_O_15_Na^+^
19	DGDG 34∶5	C 18∶3/16∶2	(+)	[M + Na]^+^	935	933.5551	C_49_H_82_O_1_5Na^+^
20	DGDG 34∶4	C 18∶4/16∶0, C18∶3/16∶1, C18∶2.16∶2, C18∶1/16∶3	(+)	[M + Na]^+^	936	935.5708	C_49_H_84_O_15_Na^+^
21	DGDG 34∶3	C 18∶3/16∶0	(+)	[M + Na]^+^	938	937.5864	C_49_H_86_O_15_Na^+^
22	DGDG 34∶2	C 18∶2/16∶0	(+)	[M + Na]^+^	940	939.6021	C_49_H_88_O_15_Na^+^
23	SQDG 32∶0	C 16∶0/16∶0	(−)	[M – H]^−^	794	793.5136	C_41_H_77_O_12_ S^−^
24	SQDG 34∶3	C 18∶3/16∶0	(−)	[M – H]^−^	816	815.4972	C_43_H_75_O_12_ S^−^
25	SQDG 34∶2	C 18∶2/16∶0	(−)	[M – H]^−^	818	817.5136	C_43_H_77_O_12_ S^−^
26	SQDG 34∶1	C 18∶1/16∶0	(−)	[M – H]^−^	820	819.5292	C_43_H_79_O_12_ S^−^
27	PI 34∶1	C 18∶1/16∶0	(−)	[M – H]^−^	836	835.5337	C_43_H_80_O_13_ P^−^
28	PG 34∶3	C 18∶3/16∶0	(−)	[M – H]^−^	744	743.4863	C_40_H_72_O_10_ P^−^
29	PG 34∶2	C 18∶1/16∶1	(−)	[M – H]^−^	746	745.5020	C_40_H_74_O_10_ P^−^

a DGTS, diacylglyceryltrimethylhomoserine; MGDG, monogalactosyldiacylglycerol; DGDG, digalactosyldiacylglycerol; SQDG, sulfoquinovosyldiacylglycerol; PI, phosphatidylinositol; PG, phosphatidylglycerol.

The fragment at m/z 243, corresponding to the galactosyl head group of [C_9_H_16_O_6_+ Na]^+^, was used for the identification of MGDG species (Fig. S2) [7]. Tandem-MS analysis of sodiated DGDG species in the positive-ion mode led to a fragment at m/z 405, corresponding to the digalactosyl head group of [C_15_H_26_O_11_+ Na]^+^ (Fig. S3) [7]. The neutral losses of 162 Da, corresponding to the loss of the hexose moiety [C_6_H_10_O_5_] of the head group, occurred in both MGDG and DGDG. In the positive ion mode of the MS/MS spectrum, lyso-glycerolipid or dehydrated lyso-glycerolipid product ions were observed remarkably well, and therefore they were used to identify the fatty acyl groups of each lipid species.

In the negative mode, 2 of MGDG, 4 of SQDG, 1 of PI, and 2 of PG were detected (Fig. 1B, Table 1). MGDG can be detected in the positive mode, but was strongly detected in the negative mode as [M + OAc]^−^ in negative mode. On the other hand, SQDG, PI, and PG were ionized in the negative mode by forming [M − H]^−^.

The major diagnostic ion for MGDG in the negative-ion mode is m/z 253, corresponding to galactosylglycerol of [C_9_H_16_O_8_]^−^ (Fig. S4). SQDG species were identified by the fragment ion at m/z 225, corresponding to the sulfoquinovosyl head group of [C_6_H_9_O_7_S]^−^ (Fig. S5) [8]. The PI species resulted in the three common fragment ions at m/z 241, 297, and 315, corresponding to the dehydrated phosphoinositol group of [C_6_H_10_O_8_P]^−^, the epoxide anion form of the phosphoinositol head group of [C_9_H_14_O_9_P]^−^, and the dehydrated glycerophosphoinositol group of [C_9_H_16_O_10_P]^−^, respectively. A neutral loss of 180 Da, corresponding to the inositol group of [C_6_H_12_O_6_] from the head group, was also observed (Fig. S6) [24], [25]. The neutral loss of 74 Da, corresponding to the loss of a dehydrated glycerol [C_3_H_6_O_2_]^−^, was used for the identification of PG species (Fig. S7) [26].

In the negative mode, since fatty acids were ionized as carboxylate ions, they could easily be identified after fragmentation, although their positions on glycerol backbone (*sn*-1 and *sn*-2) or the *cis/trans* configuration could not be determined by this type of analysis.

### 2. Changes in Lipid Profiles under Stress Conditions

A principal component analysis (PCA) was conducted on *D. tertiolecta* samples using normalized intensities of identified lipid species. The PCA score plot showed a clear separation according to stress conditions when using the compounds detected in the positive-ion mode rather than lipid species in the negative-ion mode. In particular, samples under each stress condition were efficiently distinguished from control conditions (LLNS) at the stationary phase (Fig. S8). As a consequence, variation of the lipid composition at the stationary phase was investigated in more detail. The ratio was calculated using the normalized intensities of identified lipids under each stress condition compared to those under control conditions, and the statistical significance was tested (Table 2). The actual values of the normalized intensities of lipid species were described in Table S1.

**Table 2:** The ratio of intensity of identified lipid species in *D. teriolecta* under other culture conditions compared to that intensity of them under control condition (low light and nitrate sufficient, LLNS) at the stationary phases.

			Culture condition^a^								
			LLND			HLNS			HLND		
			12h	36h	72h	12h	36h	72h	12h	36h	72h
No.	Compounds^b^	Proposed composition									
**1**	DGTS 30∶2	C 16∶0/14∶2	1.7^ n.s.^	0.9^ n.s.^	1.4^ n.s.^	1.6^ n.s.^	0.8^ n.s.^	1.1^ n.s.^	1.5^ n.s.^	1.0^ n.s.^	1.8^ n.s.^
**2**	DGTS 32∶4	C 16∶0/16∶4	1.9^ n.s.^	1.1^ n.s.^	1.2^ n.s.^	1.1^ n.s.^	0.8^ n.s.^	0.8^ n.s.^	1.6^ n.s.^	0.6 ∇	0.7^ n.s.^
**3**	DGTS 32∶2	C 16∶0/16∶2	1.4^ n.s.^	1.3^ n.s.^	1.4^ n.s.^	1.1^ n.s.^	1.2^ n.s.^	1.1^ n.s.^	1.2^ n.s.^	1.0^ n.s.^	1.3^ n.s.^
**4**	DGTS 34∶4	C 18∶4/16∶0	1.4^ n.s.^	0.5 ∇	0.6^ n.s.^	1.3^ n.s.^	0.8 ∇	0.8^ n.s.^	1.7^ n.s.^	0.3 ∇	0.5^ n.s.^
**5**	DGTS 34∶3	C 18∶3/16∶0	2.0^ n.s.^	1.0^ n.s.^	1.2^ n.s.^	1.7^ n.s.^	1.1^ n.s.^	1.1^ n.s.^	1.7^ n.s.^	0.7^ n.s.^	1.2^ n.s.^
**6**	DGTS 34∶2	C 18∶2/16∶0	2.8^ n.s.^	2.1 ▴	1.8^ n.s.^	1.9^ n.s.^	1.4^ n.s.^	1.4^ n.s.^	2.7^ n.s.^	2.0 ▴	2.7 ▴
**7**	DGTS 34∶1	C 18∶1/16∶0	2.6^ n.s.^	2.0 ▴	1.5^ n.s.^	1.7^ n.s.^	1.3^ n.s.^	1.1^ n.s.^	2.4^ n.s.^	1.9 ▴	2.1 ▴
**8**	DGTS 36∶6	C 18∶3/18∶3	1.7^ n.s.^	0.6 ∇	0.9^ n.s.^	1.1^ n.s.^	0.6 ∇	0.7^ n.s.^	1.7^ n.s.^	0.3 ∇	0.7^ n.s.^
**9**	DGTS 36∶5	C 18∶2/18∶3	2.0^ n.s.^	0.8^ n.s.^	1.3^ n.s.^	1.6^ n.s.^	0.8^ n.s.^	0.9^ n.s.^	2.1^ n.s.^	0.4 ∇	1.3^ n.s.^
**10**	DGTS 36∶4	C 18∶2/18∶2	2.2^ n.s.^	1.0^ n.s.^	1.2^ n.s.^	1.9^ n.s.^	1.0^ n.s.^	0.9^ n.s.^	2.0^ n.s.^	0.6 ∇	1.5 ▴
**11**	DGTS 36∶3	C 18∶1/18∶2	1.2^ n.s.^	0.9^ n.s.^	1.0^ n.s.^	0.9^ n.s.^	0.7^ n.s.^	0.6^ n.s.^	1.0^ n.s.^	0.5 ∇	0.9^ n.s.^
**12**	DGTS 36∶2	C 18∶1/18∶1, C18∶2/C18∶0	0.9^ n.s.^	1.0^ n.s.^	1.1^ n.s.^	0.9^ n.s.^	0.9^ n.s.^	0.8^ n.s.^	0.8^ n.s.^	0.8^ n.s.^	1.0^ n.s.^
**13**	LysoDGTS 16∶0	C 16∶0	1.1^ n.s.^	1.1^ n.s.^	0.9^ n.s.^	1.2^ n.s.^	1.3^ n.s.^	1.0^ n.s.^	0.8^ n.s.^	0.9^ n.s.^	0.9^ n.s.^
**14**	LysoDGTS 18∶3	C 18∶3	1.1^ n.s.^	0.8^ n.s.^	0.6^ n.s.^	1.2^ n.s.^	1.0^ n.s.^	0.7^ n.s.^	1.0^ n.s.^	0.7^ n.s.^	0.5 ∇
**15**	MGDG 34∶7	C 18∶3/16∶4	0.4 ^n.s^	1.3^ n.s.^	0.8^ n.s.^	0.4^ n.s.^	1.0^ n.s.^	0.5^ n.s.^	0.5^ n.s.^	1.0^ n.s.^	0.6^ n.s.^
			0.8^ n.s.^	1.0^ n.s.^	1.0^ n.s.^	1.0^ n.s.^	0.6^ n.s.^	0.9^ n.s.^	0.8^ n.s.^	0.8^ n.s.^	1.0^ n.s.^
**16**	MGDG 34∶6	C 18∶3/16∶3	0.7^ n.s.^	1.0^ n.s.^	1.1^ n.s.^	1.1^ n.s.^	0.9^ n.s.^	1.1^ n.s.^	1.0^ n.s.^	1.0^ n.s.^	1.3^ n.s.^
**17**	DGDG 34∶7	C 18∶3/16∶4	0.8^ n.s.^	1.7^ n.s.^	1.7 ▴	0.9^ n.s.^	1.5^ n.s.^	1.3^ n.s.^	0.8^ n.s.^	1.9^ n.s.^	1.6 ▴
**18**	DGDG 34∶6	C 18∶3/16∶3	0.7^ n.s.^	1.7^ n.s.^	1.7 ▴	0.8^ n.s.^	1.5^ n.s.^	1.4 ▴	0.7^ n.s.^	2.1^ n.s.^	1.9 ▴
**19**	DGDG 34∶5	C 18∶3/16∶2	0.7^ n.s.^	2.2^ n.s.^	1.8 ▴	0.8^ n.s.^	1.5^ n.s.^	1.4 ▴	0.9^ n.s.^	3.0 ▴	2.5 ▴
**20**	DGDG 34∶4	C 18∶4/16∶0, C18∶3/16∶1, C18∶2.16∶2, C18∶1/16∶3	0.7^ n.s.^	2.0^ n.s.^	1.7 ▴	0.7^ n.s.^	1.4^ n.s.^	1.3 ▴	0.9^ n.s.^	2.7 ▴	2.1 ▴
**21**	DGDG 34∶3	C 18∶3/16∶0	0.7^ n.s.^	1.1^ n.s.^	1.0^ n.s.^	0.6^ n.s.^	0.8^ n.s.^	0.8 ∇	0.7^ n.s.^	1.1^ n.s.^	0.9^ n.s.^
**22**	DGDG 34∶2	C 18∶2/16∶0	0.7^ n.s.^	0.8^ n.s.^	0.7^ n.s.^	0.6^ n.s.^	0.6 ∇	0.7^ n.s.^	0.7^ n.s.^	0.6 ∇	0.5^ n.s.^
**23**	SQDG 32∶0	C 16∶0/16∶0	0.9^ n.s.^	0.9^ n.s.^	0.9^ n.s.^	1.5^ n.s.^	1.1^ n.s.^	1.4^ n.s.^	1.2^ n.s.^	1.3 ▴	1.6^ n.s.^
**24**	SQDG 34∶3	C 18∶3/16∶0	1.0^ n.s.^	0.8^ n.s.^	0.7^ n.s.^	1.4^ n.s.^	0.9^ n.s.^	1.0^ n.s.^	0.9^ n.s.^	0.7^ n.s.^	0.7^ n.s.^
**25**	SQDG 34∶2	C 18∶2/16∶0	0.9^ n.s.^	0.8^ n.s.^	0.9^ n.s.^	1.6^ n.s.^	1.1^ n.s.^	1.1^ n.s.^	1.3^ n.s.^	1.0^ n.s.^	1.0^ n.s.^
**26**	SQDG 34∶1	C 18∶1/16∶0	0.9^ n.s.^	1.1^ n.s.^	1.0^ n.s.^	1.8^ n.s.^	1.6^ n.s.^	1.5 ▴	1.4^ n.s.^	1.6^ n.s.^	1.3^ n.s.^
**27**	PI 34∶1	C 18∶1/16∶0	0.6^ n.s.^	1.3^ n.s.^	1.1^ n.s.^	1.2^ n.s.^	1.2^ n.s.^	1.3 ▴	1.1^ n.s.^	1.6^ n.s.^	1.6 ▴
**28**	PG 34∶3	C 18∶3/16∶0	0.7^ n.s.^	0.6^ n.s.^	0.6^ n.s.^	1.2^ n.s.^	0.6^ n.s.^	0.6^ n.s.^	0.6^ n.s.^	0.5^ n.s.^	0.6^ n.s.^
**29**	PG 34∶2	C 18∶1/16∶1	0.8^ n.s.^	0.9^ n.s.^	0.9^ n.s.^	1.4^ n.s.^	0.9^ n.s.^	1.0^ n.s.^	0.8^ n.s.^	0.9^ n.s.^	1.0^ n.s.^

a LLND, low light intensity and nitrate deficiency; HLNS, high light intensity and nitrate sufficiency; HLND, high light intensity and nitrate deficiency.

b DGTS, diacylglyceryltrimethylhomoserine; MGDG, monogalactosyldiacylglycerol; DGDG, digalactosyldiacylglycerol; SQDG, sulfoquinovosyldiacylglycerol; PI, phosphatidylinositol; PG, phosphatidylglycerol.

▴, significantly increased level compared with control (*p*<0.05); ∇, significantly decreased level compared with control (*p*<0.05); n.s., no significant difference.

A nitrate deficiency had a greater impact on DGTS species than high light intensity (Table 2). When both stress factors were imposed on *D. tertiolecta*, DGTS species exhibited the largest changes. Nitrogen starvation brought about a strong increase in DGTS species containing monounsaturated fatty acid (MUFA) moieties, such as C18∶1, and one of the PUFAs, C18∶2, while DGTS species exhibited significant decreases in polyunsaturated fatty acid (PUFA) moieties, such as C18∶3 and C18∶4. *D. tertiolecta* cultures grown under high light attained changes in DGTS species containing C18∶3 and C18∶4 only.

*D. tertiolecta* grown under HLND showed a significant decrease in DGTS species consisting of C18 fatty acyl groups. The levels of DGTS species, which have the common C16∶0 fatty acid, showed variations depending on fatty acid moieties in other position. DGTS, whose fatty acyl group has four double bonds, such as C16∶4 and C18∶4, were reduced, while DGTS species, whose fatty acyl group includes C18∶1 and C18∶2, resulted in an increase in the intensity. The most intense variations in DGTS species was observed at 36 hours after the culture underwent stress. In addition, lyso-DGTS 16∶0 and lyso-DGTS 18∶3 were detected from *D. tertiolecta* samples. This may explain how they were the most abundant lyso form of species from among the detected DGTS.

The physiological role of DGTS has not yet been unraveled. According to Sato, it was assumed that DGTS would play a similar role within the cell to that of PC in higher plants because of the similarity in their chemical and physical characteristics, and the fact that the algae, which contains DGTS, rarely stores PC simultaneously [27]. PC acts as an acyl donor to the synthesis of triacylglycerol (TAG), catalyzed by phospholipid: diacylglycerol acyltransferase (PDAT). There are several reports indicating that many microalgae tend to accumulate TAG under nitrate deplete condition [28], [29]. DGTS has a role as a substrate for the desaturation of C18∶1 to 18∶2 and as a donor of primarily C18∶2 to other membrane lipids, such as PE [30]. It could then be hypothesized that the high level of DGTS with C18∶1 and C18∶2 is responsible for the enhancement of the biosynthesis of TAG. Moreover, as shown in Table 2, theses DGTS species commonly have C16∶0, fatty acids relating to energy storage, and concurrently is a predominant fatty acyl chain in TAG. Meanwhile, He et al mentioned that DGTS has been hypothesized to transfer fatty acids from the cytoplasm to chloroplasts [8]. Therefore, a significant reduction in DGTS species with PUFA could be related to an increment in the desaturation of glycerolipids, such as DGDG, which are present in chloroplasts. However, the hypotheses drawn above require verification through subsequent experimental testing.

The changes of DGDG species were observed under both nitrogen starvation and high light intensity. The significant increase of DGDG species containing unsaturated C16 fatty acid was found under these two stress conditions. In high light conditions, the DGDG species, which have a saturated fatty acid, C16∶0, showed a significant decline. When *D. tertiolecta* cultures were under nitrate deficiency and intense light, the high values of ratio were shown after 36 h in DGDG 34∶4 and DGDG 34∶5.

A neutral lipid, DGDG, facilitates the assembly of photosynthetic complexes and aids in the oligomerization of protein subunits. Consequently, it occupies functionally and structurally crucial sites of both the photosystem I and II (PSI and PSII) reaction centers [31], [32]. Hölzl and Dörmann reported that the head group of galactolipids interacts with photosynthetic protein complex [31]. Recently, the contribution of the fatty acyl chain composition to photosynthesis has been studied. Montero et al hypothesized that high light leads to an improvement in the photosynthetic activity, and it requires a higher membrane fluidity so that there could be an increase in glycerolipid species with desaturated fatty acyl chains [33]. This report would be in accordance with above result regarding the enhancement in DGDG species with unsaturated C16 fatty acyl chain under high light condition (Table 2).

Meanwhile, nitrate deficiency did not lead to any significant changes in SQDG species, while cultivation under a high light intensity resulted in an increase of SQDG (18∶1/16∶0) at 72 h. SQDG (16∶0/16∶0) showed a significant increment at 72 h in *D. tertiolecta* cultivated under nitrate deficiency and high light intensity. SQDG has an important role in maintaining function and structure of PSII complex, especially when the PSII is present under stress conditions such as high temperature [34]. The major fatty acyl chains of SQDG are known to include C16∶0 and C18∶1 in green algae [35] so that it might be related to the characteristic changes observed in SQDG with C16∶0 and/or C18∶1 under high light stress in our results.

PI 34∶1 was found to be increased at 72 h under HLNS and HLND conditions. PI was present at a low abundance in plants, and is related to cell signaling rather than acting as a structural component of the membrane. In particular, there are several reports that various kinds of phosphoinositides, which are generated from PI, were produced rapidly in response to osmotic, drought, and salt stresses [36]. However, to our knowledge, the alteration of PI by high light stress has not been reported yet.

MGDG and PG species did not exhibit variations under stress conditions, despite previous findings indicating that they have been found to play an essential role in photosynthesis [33].

### 3. LC-MS Analysis of Carotenoids

**Figure 2:**
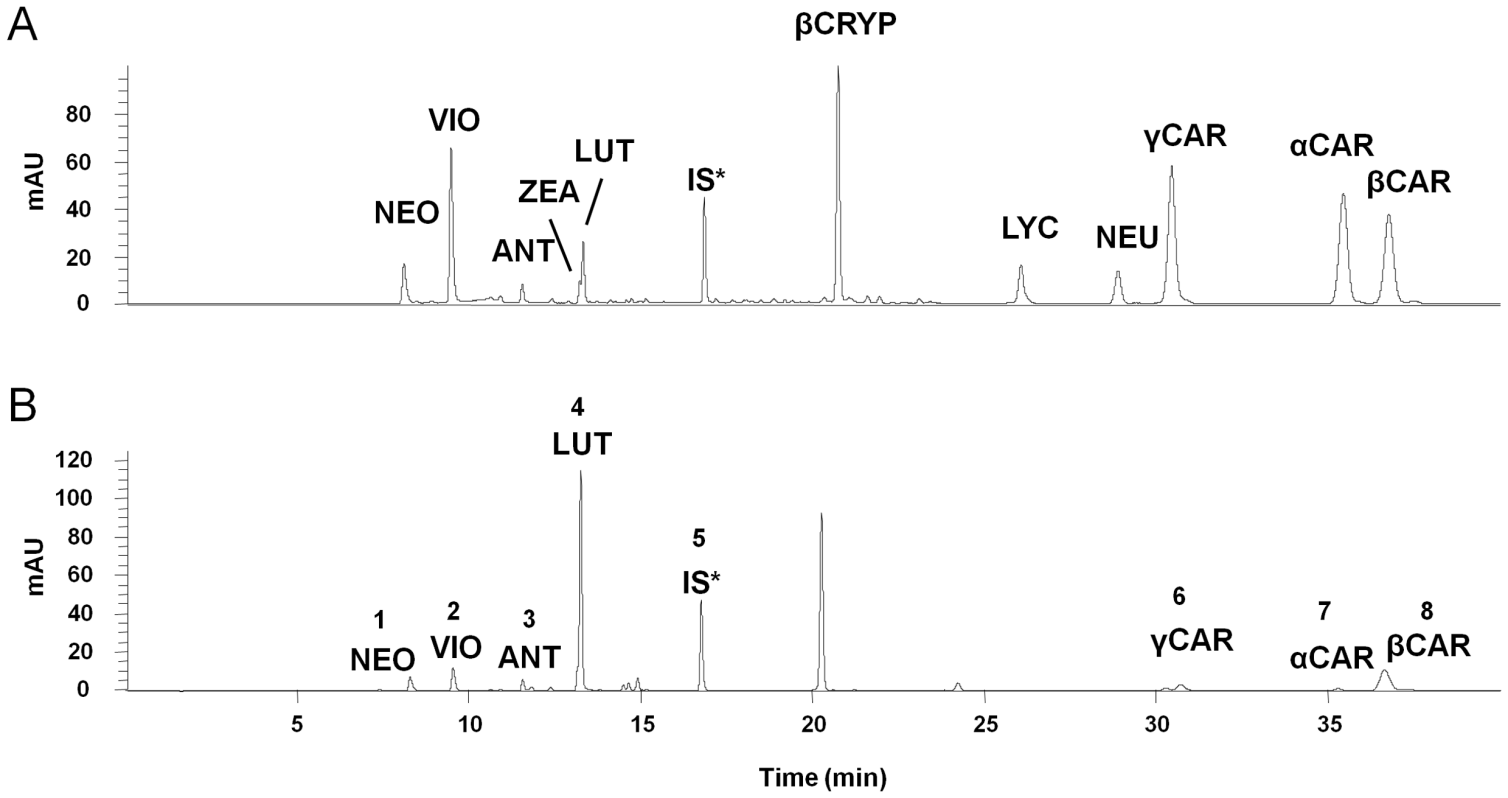
Analyses of (A) carotenoid standard mixture and (B) carotenoids extracted from *D. tertiolecta* by ultra-performance liquid chromatography (UPLC) equipped with a photodiode array (PDA) in positive-ion mode. NEO, neoxanthin; VIO, violaxanthin; ANT, anthraxanthin; ZEA, zeaxanthin; LUT, lutein; βCRYP, β-cryptoxanthin; LYC, lycopene; NEU, neurosporene; γCAR, γ-carotene; αCAR, α-carotene; βCAR, β-carotene; IS*, internal standard (β-apo-8′-carotenal). mAU, mili-absorbance unit.

Figure 2 shows the LC-MS chromatogram of carotenoids extracted from *D. tertiolecta*. Seven carotenoids were separated simultaneously within 40 min. Table 3 shows the assignment data for carotenoids in *D. tertiolecta*. All of the carotenoids ionized by APCI showed the protonated molecular ion [M + H]^+^: 601.4 for neoxanthin and violaxanthin, 585.4 for anthraxanthin, 569.4 for lutein, and 537.4 for γ-, α-, and β-carotene. The UPLC system coupled with the PDA allowed us to distinguish carotenoids species according to chromatographic retention time, although there were some carotenoids that produced an identical m/z of [M + H]^+^. The separation factors (α) and resolutions (Rs) of carotenoids in the algae for all peaks were higher than 1. Furthermore, reproducibility was good for these carotenoids as presented in the analytical results, with an relative standard deviation (R.S.D.) <0.91% for retention times and an R.S.D. <7.63% for integrated areas.

**Table 3:** List of identified carotenoids in *D. tertiolecta*.

Peak no.	Compound a	Formula	*m/z* [M + H]^+^	RT (min) b	R.S.D. RT (%)	R.S.D. area ratio (%)	α c	Rs d
**1**	NEO	C_40_H_56_O_4_	601.4	8.06	0.91	2.00	–	–
**2**	VIO	C_40_H_56_O_4_	601.4	9.43	0.58	0.99	1.20 (1, 2)^e^	3.75 (1, 2)
**3**	ANT	C_40_H_56_O_3_	585.4	11.51	0.65	1.36	1.25 (2, 3)	5.94 (2, 3)
**4**	LUT	C_40_H_56_O_2_	569.4	13.28	0.61	1.91	1.17 (3, 4)	4.72 (3, 4)
**5**	IS*			16.82	–	–	1.29 (4, 5)	10.41 (4, 5)
**6**	γCAR	C_40_H_56_	537.4	30.43	0.51	7.63	1.87 (5, 6)	18.77 (5, 6)
**7**	αCAR	C_40_H_56_	537.4	35.43	0.50	3.72	1.17 (6, 7)	5.10 (6, 7)
**8**	βCAR	C_40_H_56_	537.4	36.75	0.84	1.61	1.04 (7, 8)	1.61 (7, 8)

The mass-to-charge ratio, retention time (RT), reproducibility (R.S.D. RT and R.S.D. area ratio), separation factor (α), and resolution (Rs) were described.

a NEO, neoxanthin; VIO, violaxanthin; ANT, anthraxanthin; LUT, lutein; γCAR, γ-carotene; αCAR, α-carotene; βCAR, β-carotene; IS*, internal standard.

b Data were obtained from inter-day variability testing performed by measuring the RT and area ratio of carotenoids on the three different days, with three determinations each day, for total of nine determinations.

c Separation factor (α)  =  (RT_n+1_-RT_0_)/(RT_n_-RT_0_), where RT_n_ is the retention time of a compound, and RT_0_ is the retention time of an unretained peak.

d Rs  =  2 (RT_n+1_-RT_n_)/(W_n_+W_n+1_), where W_n_ is the band width of a compound at the baseline.

e Values in parentheses represent two neighboring peaks.

To quantify carotenoids in *D. tertiolecta*, calibration curves, the related LOD, and LOQ were obtained (Table S2). Linearity was obtained using five calibrators and three replicates. The LOD and LOQ were measured at the peak-area ratio between the analytes and internal standards against the analyte concentrations. The correlation coefficients of each calibration curve ranged from 0.9959 to 0.9999.

### 4. Changes in Carotenoid Profiles under Stress Conditions

**Figure 3:**
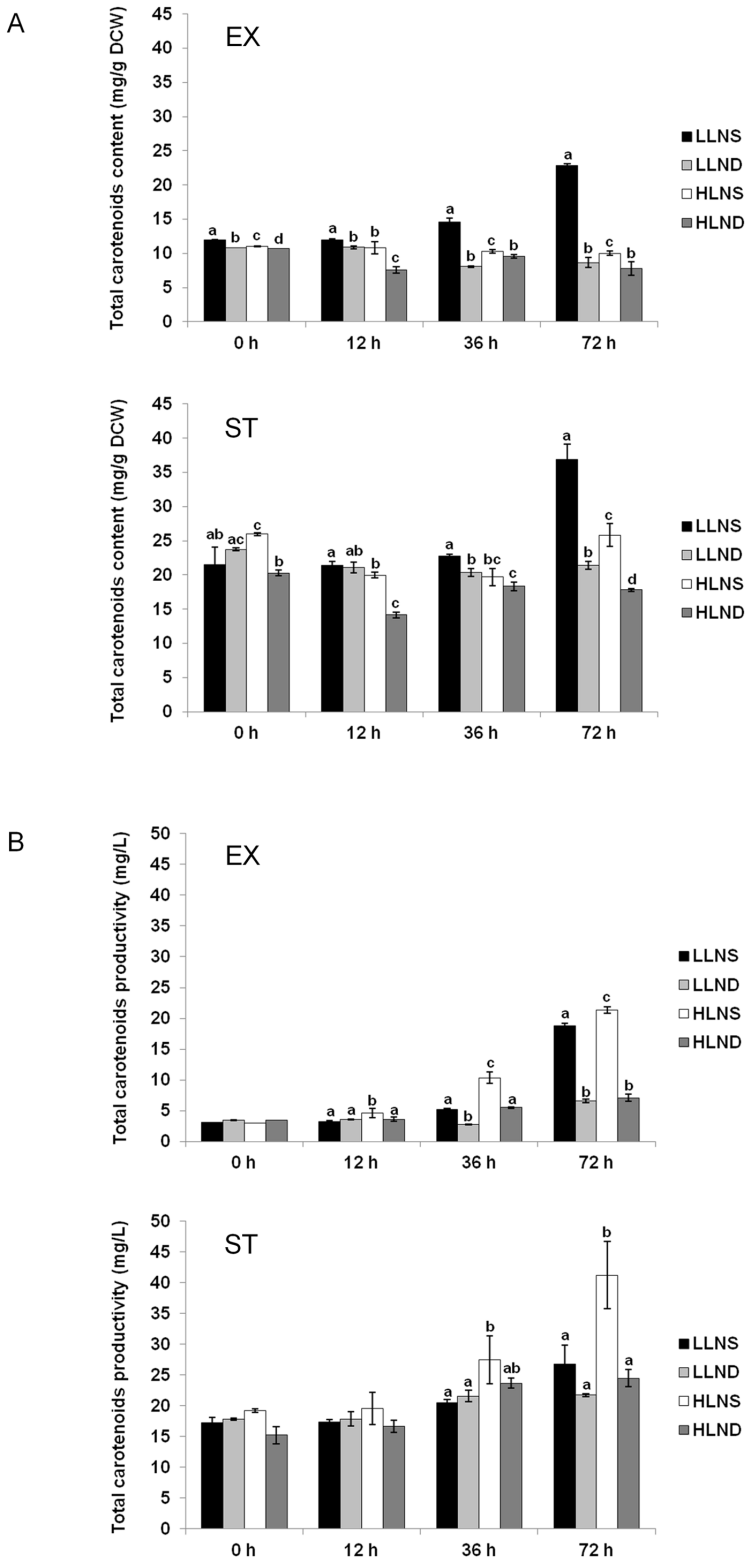
Effect of high light intensity and nitrate deficiency on the (A) total carotenoid content (mg/g dry cell weight) and (B) productivity (mg/L) of *D. tertiolecta* under shifted cultivation conditions at the exponential (EX) and stationary (ST) phases. Vertical bars represent the standard deviations (n = 4). Different alphabet in the same time point represent significant differences (*p*<0.05) within samples under various culture conditions. Sharing the same alphabet indicates no significant differences among levels in carotenoid content or productivity. LLNS, low light intensity and nitrate sufficiency; LLND, low light intensity and nitrate deficiency; HLNS, high light intensity and nitrate sufficiency; HLND, high light intensity and nitrate deficiency.

The total carotenoid content (mg/g DCW) and productivity (mg/L) are illustrated in Figure 3. The results indicated that the higher level of total carotenoid content was obtained when the stress was triggered at the stationary phase (Fig. 3A). The maximum level of total carotenoid content was showed at LLNS (control) at the stationary phase. It appeared that carotenoid accumulation under control conditions increased over time, but the carotenoid content didn't show certain tendencies under other stress conditions. The greatest enhancement of the total carotenoid content was observed in *D. tertiolecta* under LLNS followed by HLNS, LLND, and HLND at the stationary phase at 72 h (ST-72 h).

The carotenoid content of seven carotenoid species (neoxanthin, violaxanthin, anthraxanthin, lutein, and γ-, α-, and β-carotene) was analyzed and described in Table 4. The result of the changes in the carotenoid content over time and under culture conditions was similar to the total carotenoid variation, with the exception of γ-carotene. The level of γ-carotene content was increased under nitrate deficient conditions. However, the particular physiological functions of γ-carotene are not yet well understood.

**Table 4:** Time-course quantification of carotenoids^a^ in *D. tertiolecta* under the various culture conditions^b^ shifted at the exponential (EX) and stationary (ST) phases.

			0h				12h				36h				72h			
			LLNS	LLND	HLNS	HLND	LLNS	LLND	HLNS	HLND	LLNS	LLND	HLNS	HLND	LLNS	LLND	HLNS	HLND
**NEO**	EX	Content (mg/g)	1.76±0.01^a^	1.58±0.00^b^	1.73±0.01^a^	1.71±0.03^a^	1.96±0.03^a^	1.69±0.22^ab^	1.38±0.19^b^	0.94±0.08^c^	2.64±0.16^a^	1.10±0.00^bc^	1.31±0.13^b^	0.96±0.02^c^	4.09±0.06^a^	0.98±0.12^b^	1.25±0.21^b^	0.68±0.10^c^
		Productivity (mg/L)	0.45±0.00^a^	0.50±0.00^b^	0.46±0.00^a^	0.55±0.01^c^	0.52±0.04	0.55±0.07	0.59±0.13	0.44±0.05	0.95±0.04^a^	0.38±0.00^b^	1.33±0.22^c^	0.55±0.01^b^	3.36±0.02^a^	0.74±0.06 b	2.66±0.44^c^	0.61±0.07 b
	ST	Content (mg/g)	2.84±0.30^a^	3.65±0.43^b^	4.32±0.07^bc^	3.41±0.41^ab^	3.38±0.15^ab^	3.44±0.14^a^	3.14±0.10^b^	2.22±0.09^c^	4.28±0.21^a^	3.09±0.03^b^	2.75±0.28^b^	2.14±0.11^c^	7.50±0.71^a^	3.24±0.12^c^	4.25±0.59^b^	1.85±0.04^d^
		Productivity (mg/L)	2.29±0.08^a^	2.75±0.38^b^	3.19±0.06^c^	2.55±0.19^ab^	2.75±0.13	2.92±0.20	3.07±0.42	2.62±0.18	3.86±0.16^a^	3.28±0.04^ab^	3.85±0.68^a^	2.77±0.13^b^	5.44±0.84^a^	3.30±0.08^b^	6.74±0.59^ c^	2.54±0.16 b
**VIO**	EX	Content (mg/g)	0.35±0.00^a^	0.35±0.00^a^	0.39±0.01^b^	0.32±0.00^c^	0.29±0.02^a^	0.20±0.01^b^	0.38±0.01^c^	0.19±0.02^b^	0.35±0.04^a^	0.21±0.00^b^	0.35±0.02^a^	0.38±0.02^a^	0.56±0.05^a^	0.33±0.03^b^	0.49±0.02^a^	0.32±0.05^b^
		Productivity (mg/L)	0.09±0.00^a^	0.11±0.00^b^	0.10±0.00^c^	0.10±0.0^ c^	0.08±0.01^a^	0.07±0.00^a^	0.16±0.02^b^	0.09±0.01^a^	0.12±0.01^a^	0.07±0.00^b^	0.35±0.01^c^	0.22±0.01^d^	0.46±0.04 a	0.25±0.01 b	1.04±0.03^ c^	0.29±0.04^b^
	ST	Content (mg/g)	0.61±0.05^a^	0.52±0.01^a^	0.73±0.02^b^	0.50±0.08^a^	0.72±0.02^a^	0.66±0.02^a^	0.67±0.01^a^	0.47±0.05^b^	0.55±0.07	0.57±0.01	0.62±0.07	0.61±0.04	0.92±0.10^a^	0.54±0.07^b^	0.62±0.09^b^	0.51±0.06^b^
		Productivity (mg/L)	0.49±0.01^ab^	0.39±0.01^a^	0.54±0.01^b^	0.38±0.10^a^	0.59±0.01	0.56±0.03	0.66±0.10	0.55±0.04	0.49±0.05^a^	0.61±0.01^ab^	0.86±0.17^c^	0.79±0.06^bc^	0.67±0.11^a^	0.55±0.06^a^	0.99±0.04^b^	0.71±0.11^a^
**ANT**	EX	Content (mg/g)	0.38±0.01^a^	0.35±0.00^b^	0.35±0.00^b^	0.34±0.00^b^	0.47±0.02^a^	0.36±0.01^b^	0.48±0.03^a^	0.37±0.01^b^	0.49±0.03^a^	0.33±0.00^b^	0.43±0.03^a^	0.52±0.04^a^	0.59±0.02^a^	0.32±0.00^c^	0.37±0.04^bc^	0.38±0.02^b^
		Productivity (mg/L)	0.10±0.00^a^	0.11±0.00^b^	0.09±0.00^a^	0.11±0.00^b^	0.12±0.01^a^	0.12±0.00^a^	0.21±0.03^b^	0.17±0.01^b^	0.18±0.01^a^	0.11±0.00^b^	0.44±0.01^c^	0.30±0.02^d^	0.49±0.01^a^	0.24±0.01^b^	0.79±0.10^c^	0.34±0.01^b^
	ST	Content (mg/g)	0.68±0.07^a^	0.67±0.03^a^	0.81±0.01^b^	0.63±0.04^a^	0.69±0.01^a^	0.66±0.01^a^	0.69±0.03^a^	0.63±0.01^b^	0.67±0.02^a^	0.67±0.01^a^	0.66±0.07^a^	0.78±0.04^b^	1.09±0.07^a^	0.79±0.06^c^	0.81±0.06^bc^	0.80±0.04^c^
		Productivity (mg/L)	0.55±0.01^ab^	0.51±0.01^ab^	0.60±0.01^a^	0.48±0.08^b^	0.56±0.01^a^	0.56±0.02^a^	0.68±0.08^b^	0.74±0.01^b^	0.60±0.00^a^	0.71±0.01^a^	0.93±0.17^b^	1.01±0.05^b^	0.79±0.10^a^	0.80±0.05^a^	1.29±0.14^b^	1.11±0.11^b^
**LUT**	EX	Content (mg/g)	8.35±0.02^a^	7.47±0.04^b^	7.64±0.00^c^	7.30±0.04^d^	8.49±0.09^a^	7.54±0.05^b^	7.88±0.62^ab^	5.36±0.33^c^	10.10±0.28^a^	5.36±0.03^b^	7.05±0.13^c^	6.63±0.30^b^	14.95±0.13^a^	5.41±0.35^b^	6.27±0.13^c^	5.07±0.65^b^
		Productivity (mg/L)	2.13±0.01^a^	2.37±0.01^b^	2.03±0.00^c^	2.36±0.01^b^	2.24±0.14^a^	2.47±0.03^a^	3.37±0.56^b^	2.53±0.24^a^	3.62±0.03^a^	1.83±0.01^b^	7.09±0.61^c^	3.80±0.17^a^	12.29±0.21^a^	4.10±0.10^b^	13.36±0.23^c^	4.59±0.40^b^
	ST	Content (mg/g)	14.17±1.89^ab^	15.14±0.33^ac^	16.49±0.09^c^	12.73±0.15^b^	13.32±0.65^a^	13.22±0.53^a^	12.88±0.30^a^	8.92±0.28^b^	13.00±0.15^a^	12.76±0.45^a^	13.03±0.64^a^	11.52±0.52^b^	22.71±1.16^a^	13.26±0.36^b^	16.33±1.21^c^	10.91±0.10^d^
		Productivity (mg/L)	11.39±0.70^a^	11.40±0.04^a^	12.19±0.15^a^	9.56±0.94^b^	10.85±0.50^ab^	11.22±0.74^ab^	12.60±1.67^a^	10.51±0.64^b^	11.72±0.16^a^	13.53±0.66^ab^	18.16±2.26^c^	14.90±0.65^b^	16.43±1.80^a^	13.48±0.18^a^	26.03±3.05^b^	15.00±0.75^a^
**γCAR**	EX	Content (mg/g)	0.20±0.03	0.20±0.00	0.20±0.00	0.19±0.00	0.07±0.00^a^	0.23±0.02^b^	0.14±0.01^c^	0.17±0.01^d^	0.07±0.01^a^	0.36±0.04^b^	0.22±0.00^c^	0.30±0.07^bc^	0.59±0.07^a^	0.77±0.14^b^	0.47±0.03^a^	0.48±0.04^a^
		Productivity (mg/L)	0.05±0.01	0.06±0.00	0.05±0.00	0.06±0.00	0.02±0.00^a^	0.08±±0.01^b^	0.06±0.00^c^	0.08±0.01^b^	0.02±0.00^a^	0.12±0.01^b^	0.22±0.02^c^	0.17±±0.04^bc^	0.49±0.06^ab^	0.58±0.08^a^	1.00±0.06^c^	0.43±0.03^b^
	ST	Content (mg/g)	1.01±0.04^a^	1.24±0.15^b^	1.08±0.03^a^	0.94±±0.02^a^	1.03±0.05^a^	1.04±0.06^a^	0.73±0.03^b^	0.60±0.03^c^	1.26±±0.03^a^	1.27±0.08^a^	0.47±0.07^b^	1.16±0.02^a^	1.17±0.07^a^	1.47±0.05^b^	0.81±±0.21^c^	1.45±0.05^b^
		Productivity (mg/L)	0.81±0.03^ab^	0.93±0.09^a^	0.80±0.03^ab^	0.71±0.09^b^	0.84±0.04^ab^	0.88±0.07^a^	0.72±0.10^b^	0.70±0.06^b^	1.13±0.01^a^	1.35±0.10^b^	0.66±0.15^c^	1.50±0.03^b^	0.85±0.09 a	1.50±0.07^bc^	1.34±0.55^ab^	1.99±0.16^c^
**αCAR**	EX	Content (mg/g)	0.12±0.00	0.11±0.00	0.10±0.00	0.08±0.03	0.09±0.02^a^	0.07±0.01^a^	0.04±0.00^b^	0.03±±0.00^b^	0.10±0.00^a^	0.05±0.00^b^	0.06±0.01^b^	0.06±0.00^b^	0.15±0.00^a^	0.05±0.01^b^	0.05±0.00^b^	0.07±0.01^c^
		Productivity (mg/L)	0.03±0.00	0.04±0.00	0.03±0.00	0.02±0.01	0.02±0.01^a^	0.02±0.00^ab^	0.02±0.00^ab^	0.02±0.00^b^	0.03±±0.00^a^	0.02±0.00^b^	0.06±v0.01^c^	0.04±0.00^a^	0.12±0.00^a^	0.04±0.00^b^	0.10±0.00^c^	0.07±0.00^d^
	ST	Content (mg/g)	0.09±0.02^a^	0.12±0.01^b^	0.11±0.01^ab^	0.09±0.00^a^	0.10±0.01^a^	0.09±0.00^b^	0.06±0.00^c^	0.04±0.00^d^	0.23±0.08^a^	0.08±0.00^b^	0.09±0.00^b^	0.07±0.00^b^	0.26±0.02^a^	0.09±0.00^b^	0.10±0.00^bc^	0.08±0.00^c^
		Productivity (mg/L)	0.07±0.01^ab^	0.09±0.00^c^	0.08±0.00^ac^	0.07±0.00^b^	0.08±0.01^a^	0.08±0.00^a^	0.06±0.01^b^	0.04±0.00^c^	0.21±0.07^a^	0.09±0.00^b^	0.13±0.01^b^	0.09±0.00 b	0.19±0.03 a	0.09±0.00^b^	0.17±0.03^a^	0.11±0.00^b^
**βCAR**	EX	Content (mg/g)	0.79±0.01^a^	0.79±0.00^a^	0.63±0.00^b^	0.73±0.00^c^	0.64±0.16^ab^	0.77±0.04^a^	0.51±0.06^b^	0.50±0.03^b^	0.87±0.03^a^	0.65±0.00^b^	0.85±0.03^a^	0.74±0.05^b^	1.92±0.26^a^	0.80±0.08^b^	1.13±0.03^c^	0.80±0.09^b^
		Productivity (mg/L)	0.20±0.00^a^	0.25±0.00^b^	0.17±0.00^c^	0.24±0.00^d^	0.17±0.05^a^	0.25±0.01^ab^	0.22±0.04^ab^	0.24±0.02^b^	0.31±0.00^ab^	0.22±0.00 a	0.86±0.09^ c^	0.42±0.03 b	1.58±0.23 a	0.61±0.04^b^	2.40±0.07^c^	0.72±0.05^b^
	ST	Content (mg/g)	2.06±0.22^a^	2.38±0.13^b^	2.40±0.08^b^	1.98±0.01^a^	2.15±0.02^a^	1.95±0.03^b^	1.78±0.03^c^	1.27±0.08^d^	2.76±0.23^a^	1.94±0.06^b^	2.06±0.14^b^	2.04±0.22^b^	3.27±0.11^a^	2.01±0.04^b^	2.87±0.24^c^	2.20±0.04^d^
		Productivity (mg/L)	1.66±0.06^ab^	1.79±0.07^a^	1.77±0.07^a^	1.49±0.17^b^	1.75±0.02	1.65±0.07	1.75±0.24	1.50±0.14	2.49±0.26^ab^	2.05±0.09^a^	2.88±0.42^b^	2.64±0.29^ab^	2.37±0.21 a	2.05±0.07 a	4.66±1.18^b^	3.02±0.10^a^
**Total**	EX	Content (mg/g)	11.95±0.02^a^	10.85±0.04^b^	11.03±0.01^c^	10.68±0.01^d^	12.00±0.11^a^	10.87±0.17^b^	10.81±0.90^b^	7.56±0.46^c^	14.61±0.54^a^	8.06±0.07^b^	10.27±0.26^c^	9.59±0.26^b^	22.86±0.27^a^	8.66±0.71^b^	10.02±0.28^c^	7.79±0.96^b^
		Productivity (mg/L)	3.05±0.00^a^	3.44±0.01^b^	2.93±0.00^c^	3.45±0.00^b^	3.17±0.26^a^	3.56±0.04^a^	4.62±0.79^b^	3.56±0.34 a	5.24±0.08^a^	2.76±0.02^b^	10.34±0.96^c^	5.50±0.15 a	18.79±0.36 a	6.56±0.28 b	21.35±0.51^c^	7.06±0.58^b^
	ST	Content (mg/g)	21.46±2.58^ab^	23.72±0.21^ac^	25.95±0.23^c^	20.28±0.42^b^	21.38±0.56^a^	21.07±0.78^ab^	19.96±0.44^b^	14.14±0.43^c^	22.74±0.27^a^	20.38±0.57^b^	19.70±1.27^bc^	18.32±0.64^c^	36.92±2.18^a^	21.40±0.53^b^	25.80±1.67^c^	17.80±0.23^d^
		Productivity (mg/L)	17.26±0.83^a^	17.86±0.17^a^	19.18±0.29^a^	15.22±1.44^b^	17.43±0.41	17.87±1.13	19.54±2.61	16.66±0.99	20.51±0.48^a^	21.61±0.88^a^	27.48±3.85^b^	23.70±0.80^ab^	26.73±3.15^a^	21.76±0.24^a^	41.22±5.47^b^	24.48±1.40^a^

a NEO, neoxanthin; VIO, violaxanthin; ANT, anthraxanthin; LUT, lutein; γCAR, γ-carotene; αCAR, α-carotene; βCAR, β-carotene; Total, total carotenoids.

b LLNS, low light intensity and nitrate sufficiency; LLND, low light intensity and nitrate deficiency; HLNS, high light intensity and nitrate sufficiency; HLND, high light intensity and nitrate deficiency.

Each value represents the mean ± standard deviation (SD) (n = 4)

Different letters in the same time point represent significant differences (*p*<0.05) within samples under various culture conditions.

Among all detected carotenoids in *D. tertiolecta* cells, lutein showed the highest level of content. Although *D. salina* showed a differential response to stresses, such as different salinity levels, compared to *D. tertiolecta* [37], this result is in agreement with a previous report, in which one of the major carotenoids in *D. salina* was lutein [21]. In addition, Lamers et al reported that β-carotene accumulation in *D. salina* was induced upon the shift to an increased light intensity, from 200 to 1,400 μE/m^2^s whereas lutein concentrations decreased sharply upon light stress [38]. According to this result, it assumed that the degree of high light intensity (300 μE/m^2^s) in our study might not be enough to increase the accumulation of β-carotene. Geider et al reported that photosynthetic carotenoids (lutein, neoxanthin, violaxanthin, and antheraxanthin) were reduced by nitrogen limitation in *D. tertiolecta* [39], which coincides with our results.

The total carotenoid productivity of *D. tertiolecta* at the stationary phase was higher than in the exponential phase, and it increased gradually over time (Fig. 3B). The highest level of total carotenoid productivity was obtained in *D. tertiolecta* culture under HLNS. This result came as a result of the fact that the highest dried cell weight of *D. tertiolecta* cell was acquired under this condition (data is not shown). As shown in Table 4, the highest levels of productivity of neoxanthin, violaxanhin, anthraxanthin, lutein, and β-carotene were recorded in HLNS at ST-72 h. However, α-carotene and γ-carotene were shown to have the highest productivity in LLNS at stationary phase at 36 h (ST-36 h) and HLND at ST-72 h, respectively. With the exception of violaxanthin, the other six carotenoids showed about a two-fold increase in the productivity levels at ST-72 h than during the exponential phase at 72 h (EX-72 h) in HLNS condition. Therefore, from the industrial point of view, HLNS conditions would yield the maximum productivity of carotenoids. For the enhancement of productivity in most of the carotenoids, nitrate limitation was unnecessary, whereas γ-carotene showed the highest level of productivity in *D. tertiolecta* cell culture under high-light and nitrate-deficient conditions.

## Conclusion

NanoESI chip based direct infusion and UPLC-PDA-MS were successfully applied to elucidate changes in the lipid and carotenoid composition in *D. tertiolecta* under different nutrient-concentration and light-intensity conditions. A total of 29 lipids, including various species such as DGTS, MGDG, DGDG, SQDG, PG, and PI, were identified. Moreover, seven species of carotenoids, including neoxanthin, violaxanthin, anthraxanthin, lutein, and γ-, α-, and β-carotene, were qualified and quantified. Each lipid species showed a different variation in the fatty acid saturation, which might be related to photosynthesis in microalgae. A distinct discrimination of the lipid profiling among the stress conditions was shown when stress was induced at the stationary phase (ST) rather than during the exponential phase (EX). Also, the highest total carotenoid content and productivity were attained by inducing at the ST.

This is the first report of glycerolipid, glycerophospholipid, and carotenoid profiling in *D. tertiolecta*, and could provide new insights into the biological functions of their lipid species in response to nutrient and light stress. Moreover, this study suggested a methodology for identifying individual lipid species by using lipidomics techniques, which might contribute to providing more advanced knowledge for the enhancement of biofuel or biopharmaceutical materials production in microalgae systems.

## Supporting Information

Figure S1 **Positive-ion ESI tandem mass spectrum of [M + H]^+^ (at** ***m/z*** **734) for major diacylglyceryltrimethylhomoserine (DGTS) species (C18∶3/C16∶0-DGTS).**
(TIF)

Figure S2 **Positive-ion ESI tandem mass spectrum of [M + Na]^+^ (at** ***m/z*** **767) for major** **monogalactosyldiacylglycerol (MGDG) species (C18∶3/C16∶4-MGDG).**
(TIF)

Figure S3 **Positive-ion ESI tandem mass spectrum of [M + Na]^+^ (at** ***m/z*** **931) for major digalactosyldiacylglycerol (DGDG) species (C18∶3/C16∶3-DGDG).**
(TIF)

Figure S4 **Negative-ion ESI tandem mass spectrum of [M + OAc]^−^ (at** ***m/z*** **803) for major monogalactosyldiacylglycerol (MGDG) species (C18∶3/C16∶4-MGDG).**
(TIF)

Figure S5 **Negative-ion ESI tandem mass spectrum of [M – H]^−^ (at** ***m/z*** **815) for major sulfoquinovosyl-diacylglycerol (SQDG) species (C18∶3/C16∶0-SQDG).**
(TIF)

Figure S6 **Negative-ion ESI tandem mass spectrum of [M – H]^−^ (at** ***m/z*** **835) for major phosphatidylinositol (PI) species (C18∶1/C16∶0-PI).**
(TIF)

Figure S7 **Negative-ion ESI tandem mass spectrum of [M – H]^−^ (at** ***m/z*** **745) for major phosphatidylglycerol (PG) species (C18∶2/C16∶0-PG).**
(TIF)

Figure S8 **PCA score plot derived from ESI-MS/MS of D. tertiolecta samples in (A) positive- and (b) negative-ion modes**. EX, exponential phase; ST, stationary phase. •, LLNS; ▪, LLND; ▴, HLNS; ▾, HLND. The samples of each group are biological replicates. The time located at the bottom indicates the hours after triggering the stress condition.
(TIF)

Table S1 **The normalized ion intensities of the identified lipids over the culture conditions and time** **course in *D. tertiolecta.***
(DOCX)

Table S2 **Regression equations, correlation coefficients (r^2^ values), LOD, and LOQ of carotenoids.**
(DOCX)

## References

[1] Hosseini TA, Shariati M (2009) *Dunaliella* biotechnology: Methods and applications. J Appl Microbiol 107: 14–35. 19245408 10.1111/j.1365-2672.2009.04153.x

[2] Ben-Amotz A, Avron M (1990) The biotechnology of cultivating the halotolerant alga *Dunaliella* . Trends Biotechnol 8: 121–126.

[3] Evans R, Kates M, Ginzburg M, Ginzburg B (1982) Lipid composition of halotolerant algae, *Dunaliella parva* lerche *and Dunaliella tertiolecta* . 712: 186–195.

[4] Barzegari A, Hejazi MA, Hosseinzadeh N, Eslami S, Mehdizadeh AE, et al (2010) *Dunaliella* as an attractive candidate for molecular farming. Mol Biol Rep 37: 3427–3430. 19943116 10.1007/s11033-009-9933-4

[5] Tang H, Abunasser N, Garcia M, Chen M, Simon Ng KY, et al (2011) Potential of microalgae oil from *Dunaliella tertiolecta*as a feedstock for biodiesel. Appl Energy 88: 3324–3330.

[6] Chen M, Tang H, Ma H, Holland TC, Simon Ng KY, et al (2011) Effect of nutrients on growth and lipid accumulation in the green algae *Dunaliella tertiolecta* . Bioresour Technol 102: 1649–1655. 20947341 10.1016/j.biortech.2010.09.062

[7] Lu N, Wei D, Chen F, Yang S (2012) Lipidomic profiling and discovery of lipid biomarkers in snow alga *Chlamydomonas nivalis* under salt stress. Eur J Lipid Sci Technol 114: 253–265.

[8] He H, Rodgers RP, Marshall AG, Hsu CS (2011) Algae polar lipids characterized by online liquid chromatography coupled with hybrid linear quadrupole ion Trap/Fourier transform ion cyclotron resonance mass spectrometry. Energy Fuels 25: 4770–4775.

[9] Kind T, Meissen JK, Yang D, Nocito F, Vaniya A, et al (2012) Qualitative analysis of algal secretions with multiple mass spectrometric platforms. J Chromatogr A 1244: 139–147. 22608776 10.1016/j.chroma.2012.04.074 PMC3746802

[10] Fan J, Andre C, Xu C (2011) A chloroplast pathway for the de novo biosynthesis of triacylglycerol in *Chlamydomonas reinhardtii* . FEBS Lett 585: 1985–1991. 21575636 10.1016/j.febslet.2011.05.018

[11] Touchstone JC (1995) Thin-layer chromatographic procedures for lipid separation. J Chromatogr B Biomed Appl 671: 169–195. 8520691 10.1016/0378-4347(95)00232-8

[12] Hu C, van der Heijden R, Wang M, van der Greef J, Hankemeier T, et al (2009) Analytical strategies in lipidomics and applications in disease biomarker discovery. J Chromatogr B Analyt Technol Biomed Life Sci 877: 2836–2846. 10.1016/j.jchromb.2009.01.038 19233743

[13] Goss R, Wihelm C (2009) Lipids in algae, lichens and mosses. In: Wada H, Murata N, editors. Lipids in photosynthesis: essential and regulatory functions. Dordrecht: Springer. pp. 117–137.

[14] Guillard RRL (1975) Culture of phytoplankton for feeding marine invertebrates. In: Smith WL, Chanley MH, editors. Culture of marine invertebrate animals. New York: Plenum Press. pp. 26–60.

[15] Choi S, Suh IS, Lee C (2003) Lumostatic operation of bubble column photobioreactors for *Haematococcus pluvialis* cultures using a specific light uptake rate as a control parameter. Enzyme Microb Technol 33: 403–409.

[16] Lee HS, Seo MW, Kim ZH, Lee CG (2006) Determining the best specific light uptake rates for the lumostatic cultures in bubble column photobioreactors. Enzyme Microb Technol 39: 447–452.

[17] Folch J, Lees M, Sloane-Stanley G (1957) A simple method for the isolation and purification of total lipids from animal tissues. J Biol Chem 226: 497–509. 13428781

[18] Bohoyo-Gil D, Dominguez-Valhondo D, García-Parra J, González-Gómez D (2012) UHPLC as a suitable methodology for the analysis of carotenoids in food matrix. Eur Food Res Technol 235: 1055–1061.

[19] Scaife MA, Ma CA, Armenta RE (2012) Efficient extraction of canthaxanthin from *Escherichia coli* by a 2-step process with organic solvents. Bioresour Technol 111: 276–281. 22353211 10.1016/j.biortech.2012.01.155

[20] Rivera S, Vilaró F, Canela R (2011) Determination of carotenoids by liquid chromatography/mass spectrometry: effect of several dopants. Anal Bioanal Chem 400: 1339–1346. 21380750 10.1007/s00216-011-4825-6

[21] Fu W, Magnúsdóttir M, Brynjólfson S, Palsson BØ, Paglia G (2012) UPLC-UV-MS(E) analysis for quantification and identification of major carotenoid and chlorophyll species in algae. Anal Bioanal Chem 404: 3145–3154. 23052878 10.1007/s00216-012-6434-4

[22] Kaiser P, Geyer R, Surmann P, Fuhrmann H (2011) LC-MS method for screening unknown microbial carotenoids and isoprenoid quinones. J Microbiol Methods 88: 28–34. 22036764 10.1016/j.mimet.2011.10.001

[23] Lee M, Chen B (2001) Separation of lycopene and its cis isomers by liquid chromatography. Chromatographia 54: 613–617.

[24] Pulfer M, Murphy RC (2003) Electrospray mass spectrometry of phospholipids. Mass Spectrom Rev 22: 332–364. 12949918 10.1002/mas.10061

[25] Vallejo MC, Nakayasu ES, Longo LV, Ganiko L, Lopes FG, et al (2012) Lipidomic analysis of extracellular vesicles from the pathogenic phase of paracoccidioides brasiliensis. PLoS One 7: e39463. 22745761 10.1371/journal.pone.0039463 PMC3382159

[26] Larsen Å, Uran S, Jacobsen PB, Skotland T (2001) Collision–induced dissociation of glycero phospholipids using electrospray ion–trap mass spectrometry. Rapid Commun Mass Spectrom 15: 2393–2398. 11746908 10.1002/rcm.520

[27] Sato N (1992) Betaine lipids. J Plant Res 105: 185–197.

[28] Tornabene T, Holzer G, Lien S, Burris N (1983) Lipid composition of the nitrogen starved green alga *Neochloris oleoabundans* . Enzyme Microb Technol 5: 435–440.

[29] Hu Q, Sommerfeld M, Jarvis E, Ghirardi M, Posewitz M, et al (2008) Microalgal triacylglycerols as feedstocks for biofuel production: perspectives and advances. Plant J 54: 621–639. 18476868 10.1111/j.1365-313X.2008.03492.x

[30] Vogel G, Eichenberger W (1992) Betaine lipids in lower plants. biosynthesis of DGTS and DGTA in *Ochromonas danica* (*Chrysophyceae*) and the possible role of DGTS in lipid metabolism. Plant Cell Physiol 33: 427–436.

[31] Hölzl G, Dörmann P (2007) Structure and function of glycoglycerolipids in plants and bacteria. Prog Lipid Res 46: 225–243. 17599463 10.1016/j.plipres.2007.05.001

[32] Sozer O, Kis M, Gombos Z, Ughy B (2011) Proteins, glycerolipids and carotenoids in the functional photosystem II architecture. Front Biosci 16: 619–643. 10.2741/3710 21196193

[33] Montero O, Sanchez-Guijo A, Lubian LM, Martinez-Rodriguez G (2012) Changes in membrane lipids and carotenoids during light acclimation in a marine cyanobacterium *Synechococcus* sp. J Biosci 37: 635–645. 22922189 10.1007/s12038-012-9234-2

[34] Sato N (2004) Roles of the acidic lipids sulfoquinovosyl diacylglycerol and phosphatidylglycerol in photosynthesis: their specificity and evolution. J Plant Res 117: 495–505. 15538651 10.1007/s10265-004-0183-1

[35] Khotimchenko S (2002) Distribution of glyceroglycolipids in marine algae and grasses. Chem Nat Compd 38: 223–229.

[36] Zhu JK (2002) Salt and drought stress signal transduction in plants. Annu Rev Plant Biol 53: 247–273. 12221975 10.1146/annurev.arplant.53.091401.143329 PMC3128348

[37] Tammam AA, Fakhry EM, El-Sheekh M (2011) Effect of salt stress on antioxidant system and the metabolism of the reactive oxygen species in *Dunaliella salina* and *Dunaliella tertiolecta* . Afr J Biotechnol 10: 3795–3808.

[38] Lamers PP, van de Laak, Carlien CW, Kaasenbrood PS, Lorier J, et al (2010) Carotenoid and fatty acid metabolism in light-stressed *Dunaliella salina* . Biotechnol Bioeng 106: 638–648. 20229508 10.1002/bit.22725

[39] Geider R, Macintyre, Graziano L, McKay RM (1998) Responses of the photosynthetic apparatus of *Dunaliella tertiolecta* (*chlorophyceae*) to nitrogen and phosphorus limitation. Eur J Phycol 33: 315–332.

