# Supplementary figures and images for: Effects of Light Intensity and Nitrogen Starvation on Glycerolipid, Glycerophospholipid, and Carotenoid Composition in Dunaliella tertiolecta Culture
Source: PLoS One. 2013 Sep 5;8(9):e72415. doi: 10.1371/journal.pone.0072415 (PMC3764108; doi:10.1371/journal.pone.0072415)

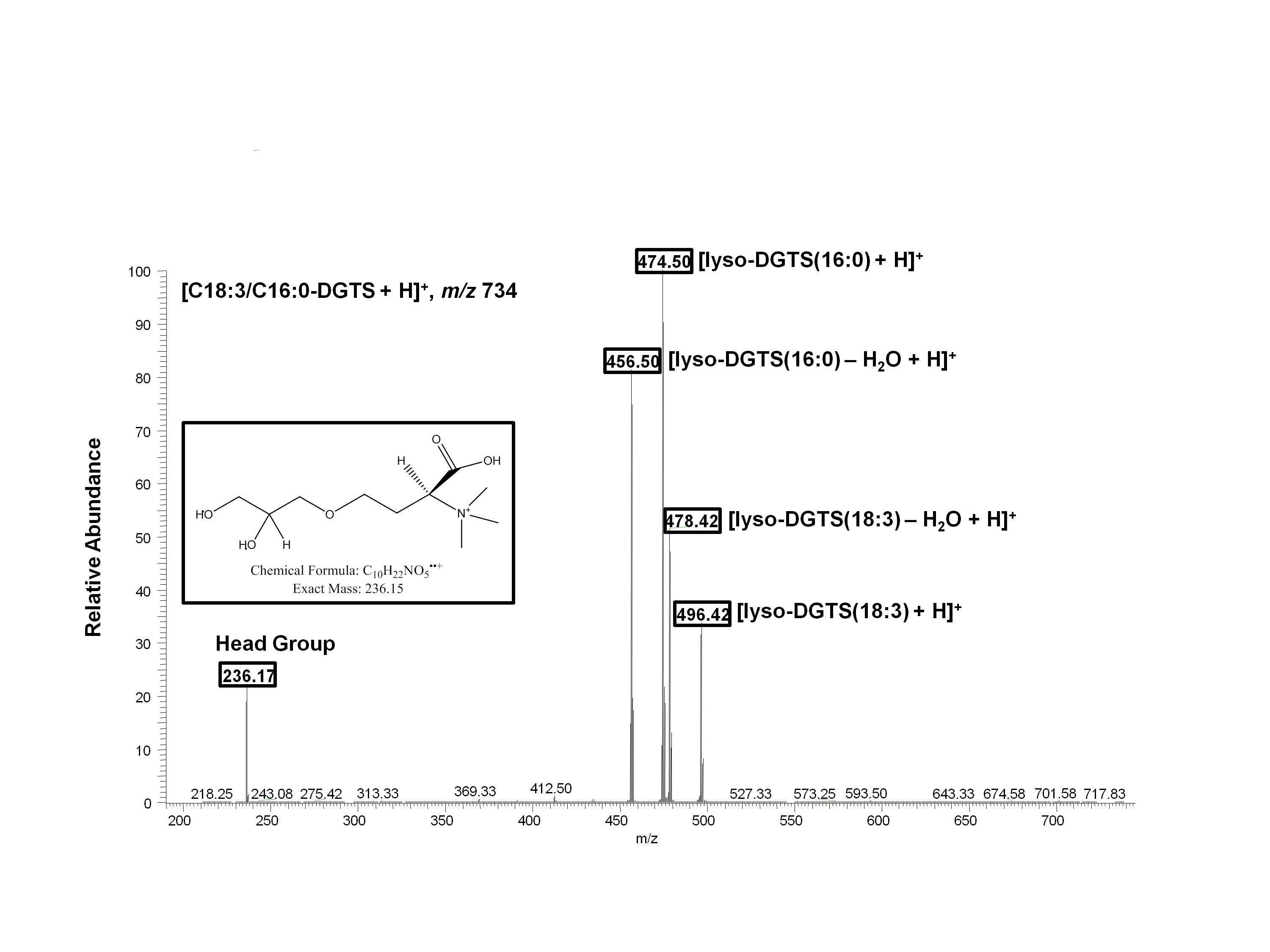

Supplement: Figure S1 — Positive-ion ESI tandem mass spectrum of [M + H]+ (at m/z 734) for major diacylglyceryltrimethylhomoserine (DGTS) species (C18∶3/C16∶0-DGTS). (TIF) [file pone.0072415.s001.tif]

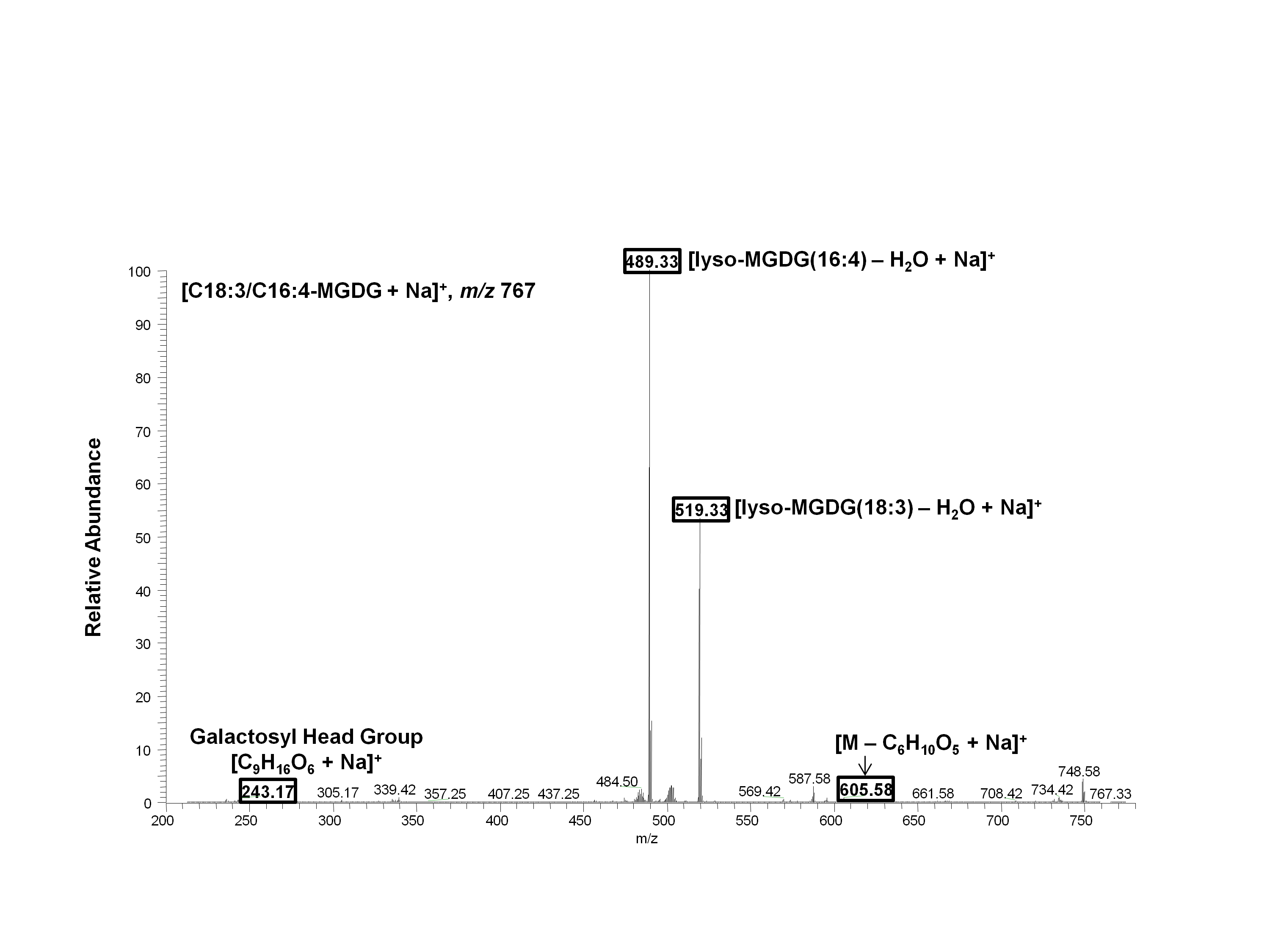

Supplement: Figure S2 — Positive-ion ESI tandem mass spectrum of [M + Na]+ (at m/z 767) for major monogalactosyldiacylglycerol (MGDG) species (C18∶3/C16∶4-MGDG). (TIF) [file pone.0072415.s002.tif]

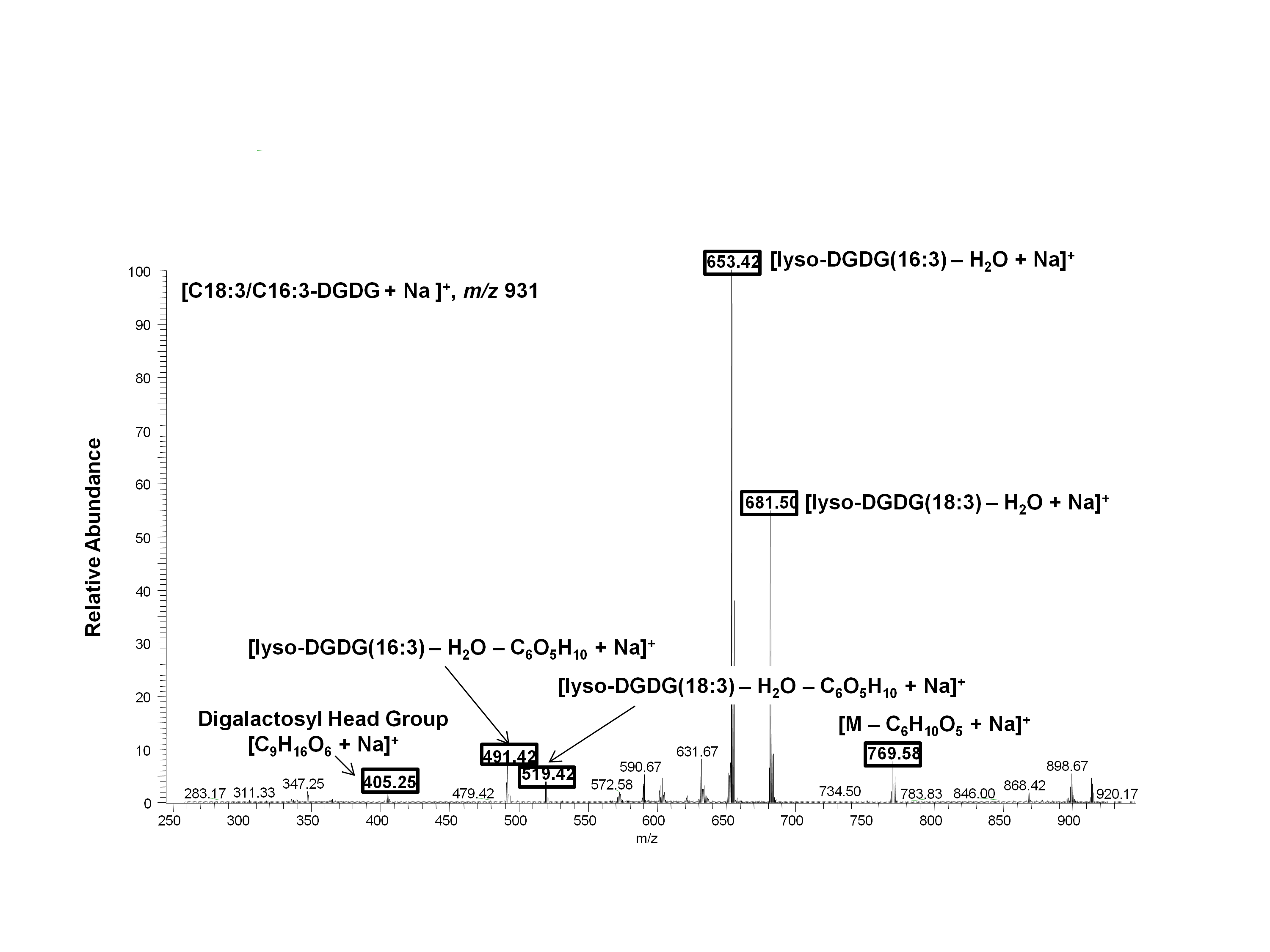

Supplement: Figure S3 — Positive-ion ESI tandem mass spectrum of [M + Na]+ (at m/z 931) for major digalactosyldiacylglycerol (DGDG) species (C18∶3/C16∶3-DGDG). (TIF) [file pone.0072415.s003.tif]

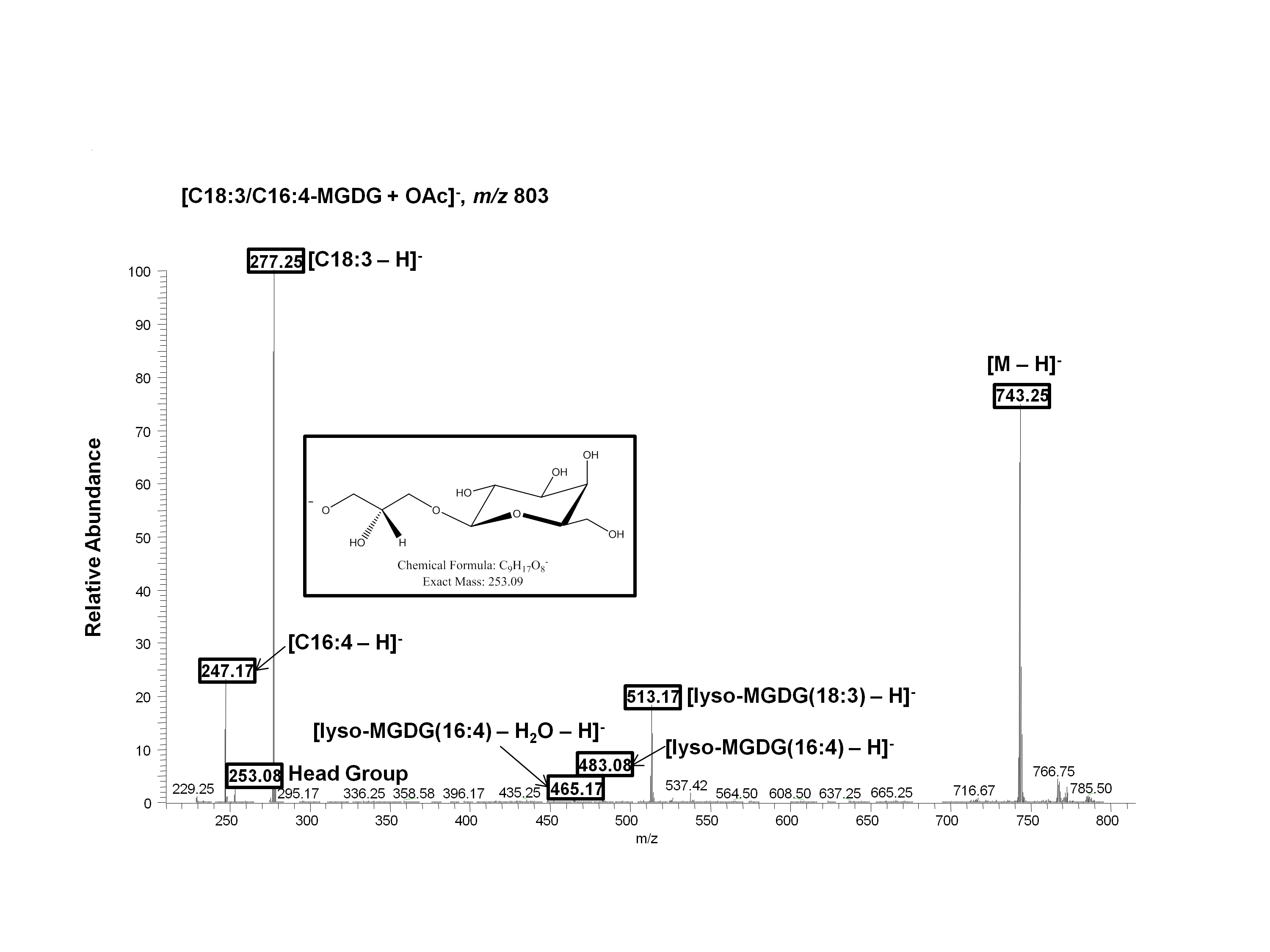

Supplement: Figure S4 — Negative-ion ESI tandem mass spectrum of [M + OAc]− (at m/z 803) for major monogalactosyldiacylglycerol (MGDG) species (C18∶3/C16∶4-MGDG). (TIF) [file pone.0072415.s004.tif]

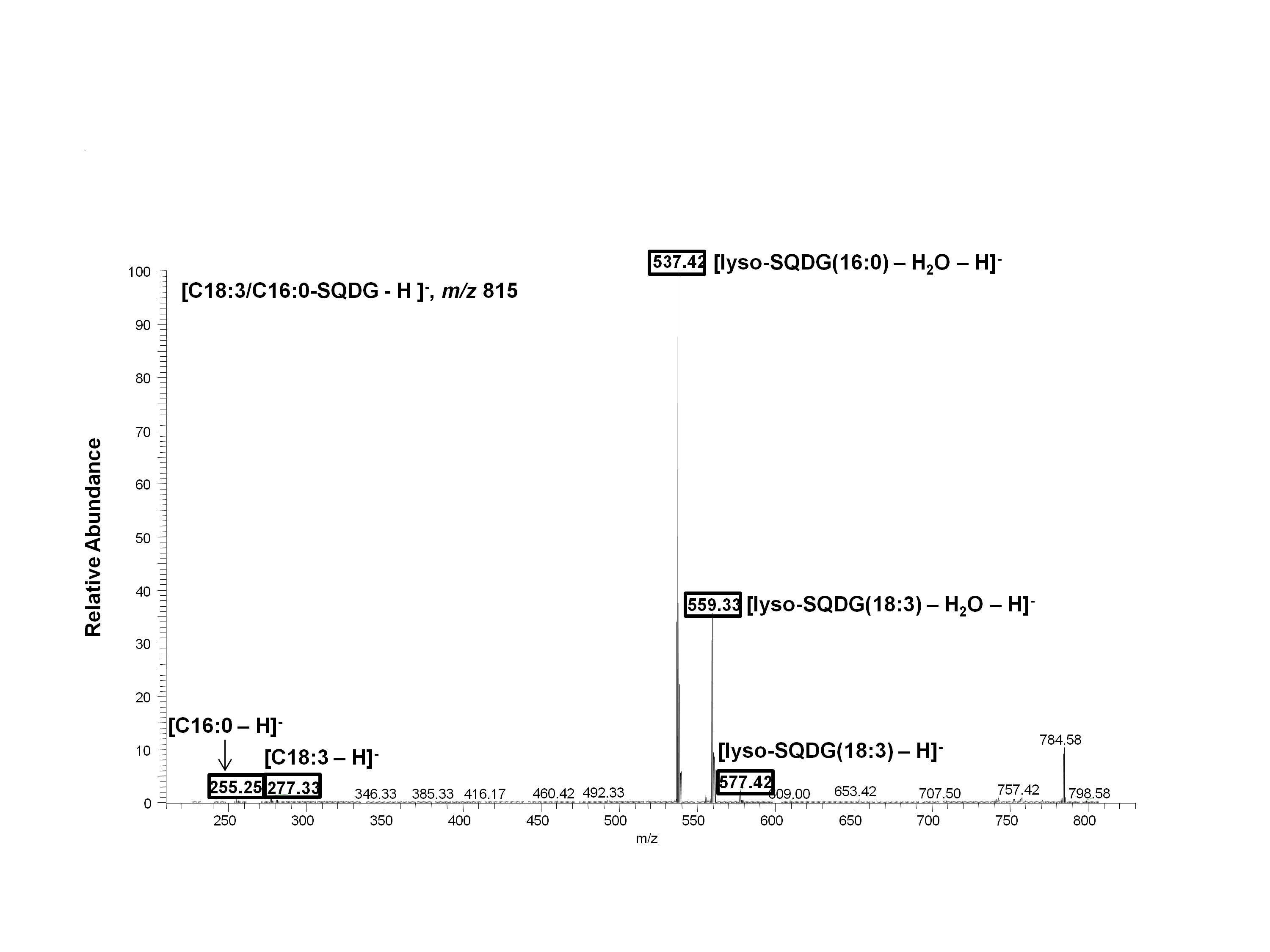

Supplement: Figure S5 — Negative-ion ESI tandem mass spectrum of [M – H]− (at m/z 815) for major sulfoquinovosyl-diacylglycerol (SQDG) species (C18∶3/C16∶0-SQDG). (TIF) [file pone.0072415.s005.tif]

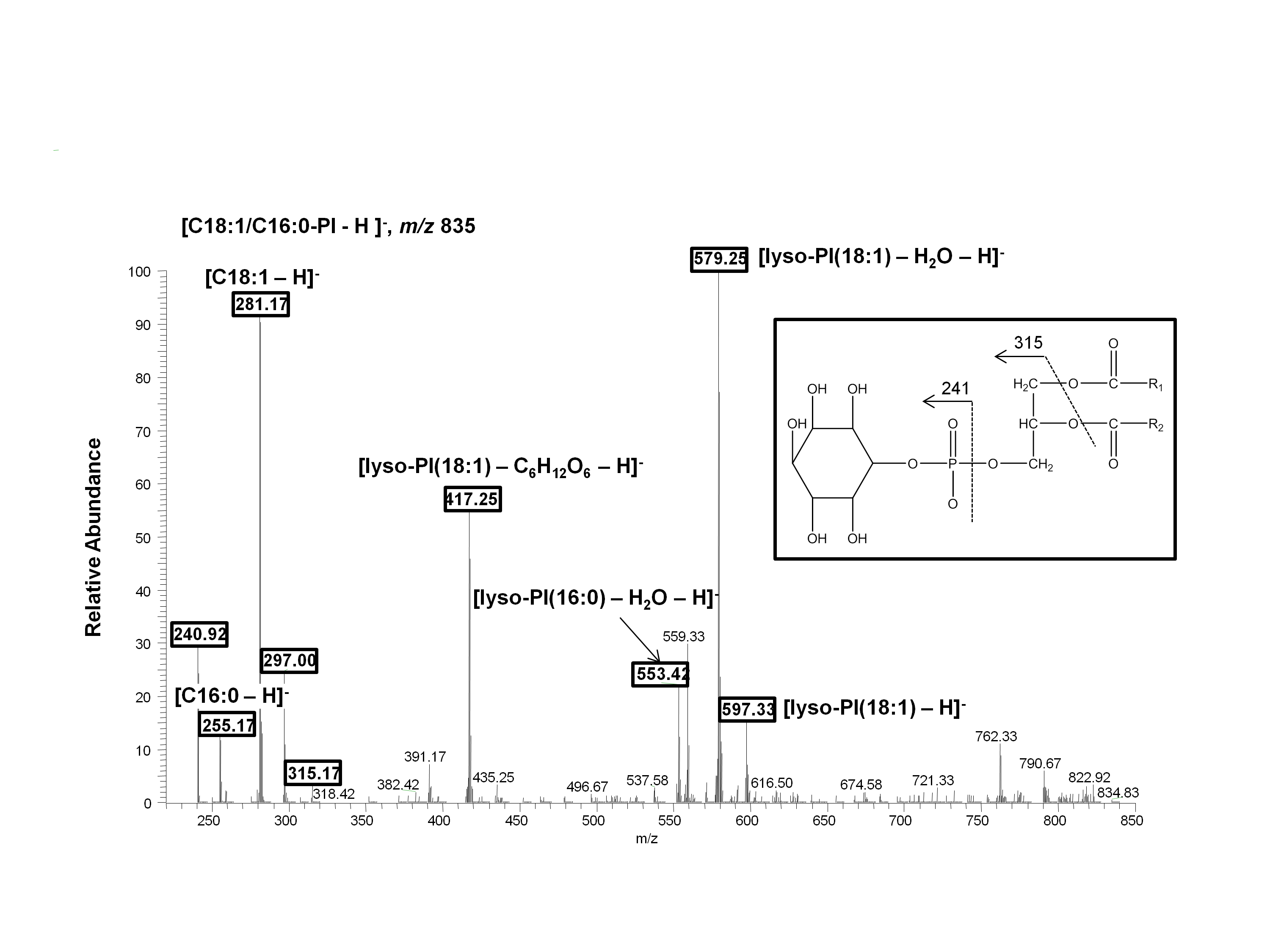

Supplement: Figure S6 — Negative-ion ESI tandem mass spectrum of [M – H]− (at m/z 835) for major phosphatidylinositol (PI) species (C18∶1/C16∶0-PI). (TIF) [file pone.0072415.s006.tif]

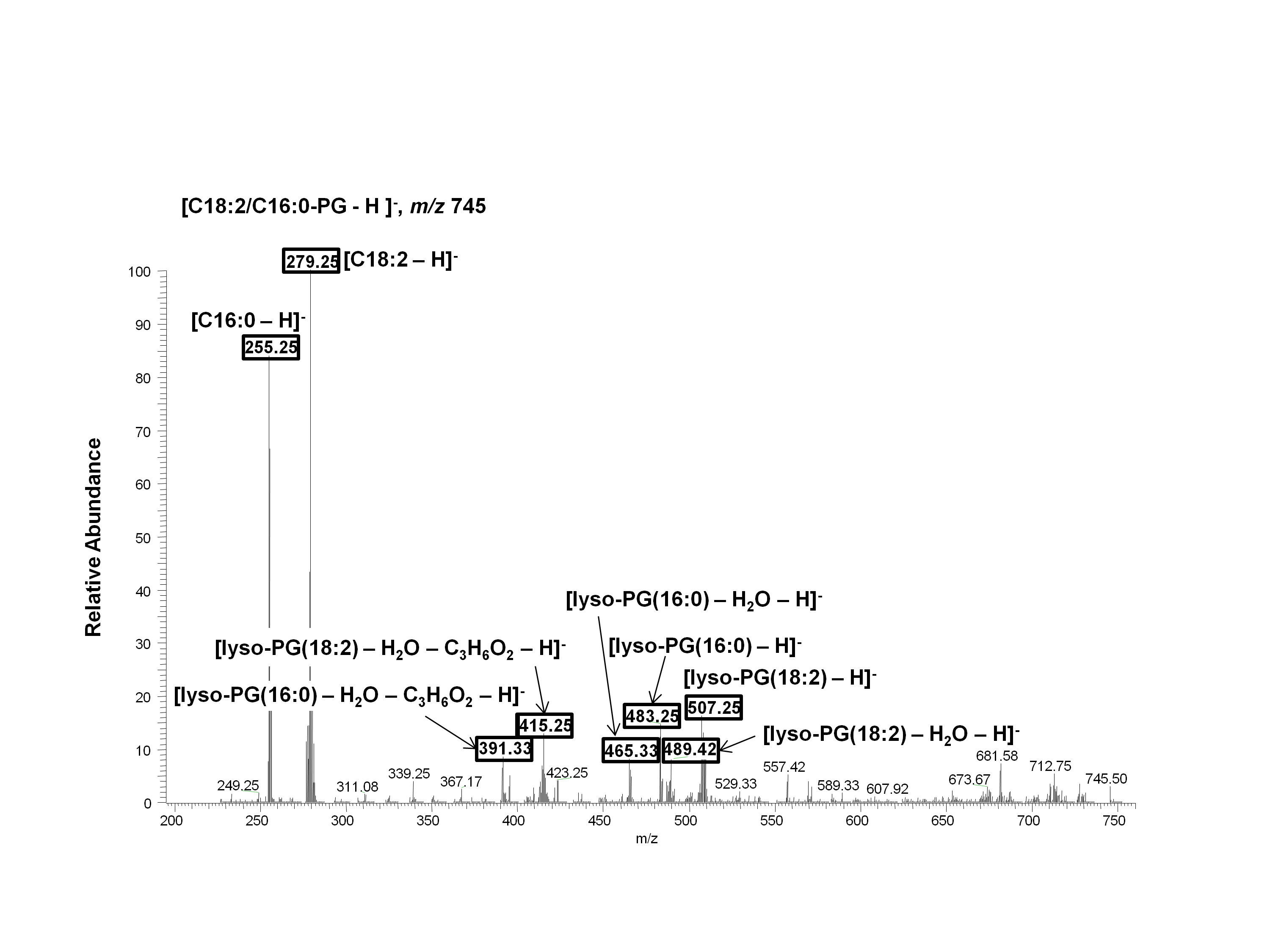

Supplement: Figure S7 — Negative-ion ESI tandem mass spectrum of [M – H]− (at m/z 745) for major phosphatidylglycerol (PG) species (C18∶2/C16∶0-PG). (TIF) [file pone.0072415.s007.tif]

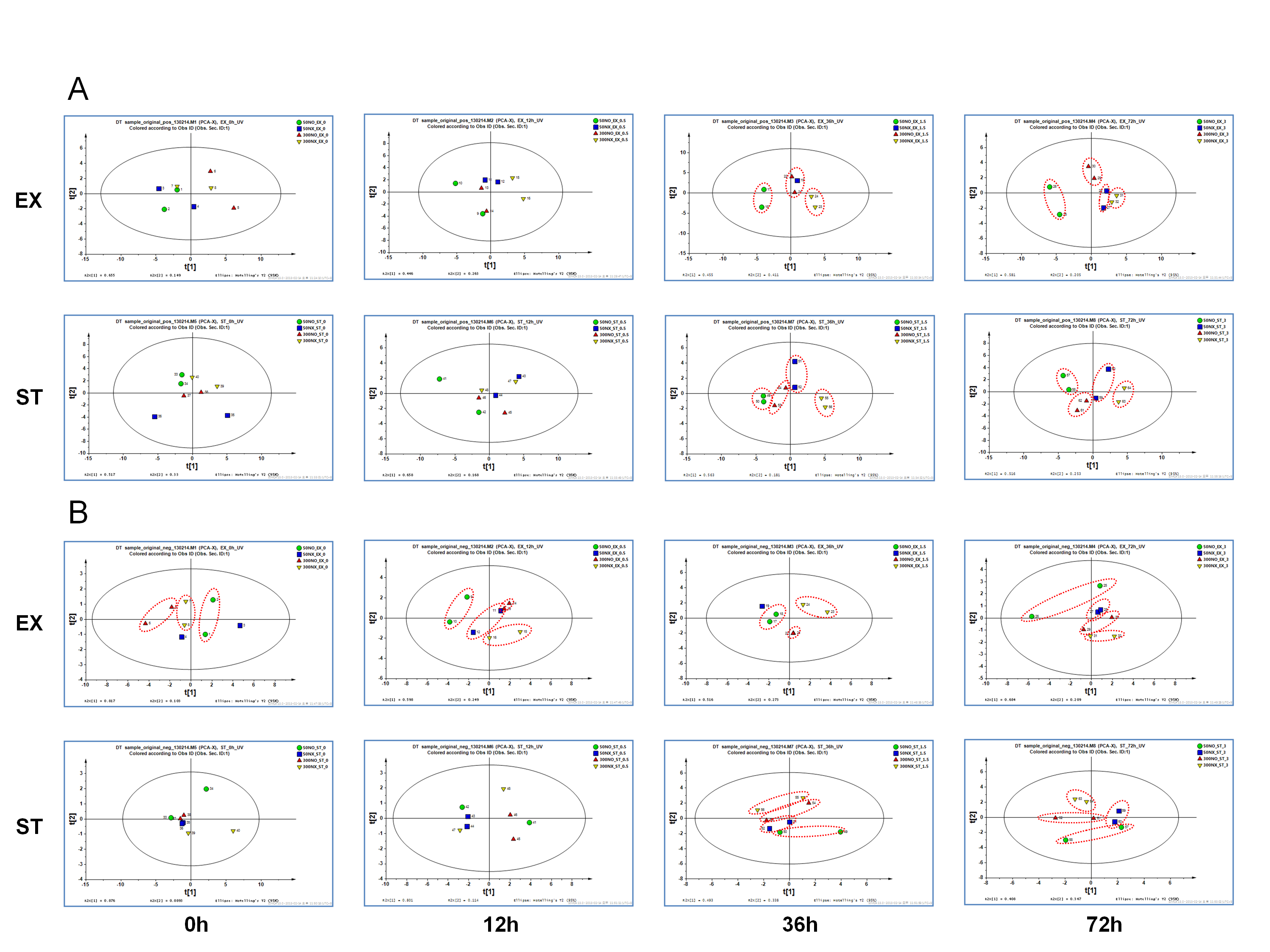

Supplement: Figure S8 — PCA score plot derived from ESI-MS/MS of D. tertiolecta samples in (A) positive- and (b) negative-ion modes. EX, exponential phase; ST, stationary phase. •, LLNS; ▪, LLND; ▴, HLNS; ▾, HLND. The samples of each group are biological replicates. The time located at the bottom indicates the hours after triggering the stress condition. (TIF) [file pone.0072415.s008.tif]
