# Supplementary material for: Effects of Light Intensity and Nitrogen Starvation on Glycerolipid, Glycerophospholipid, and Carotenoid Composition in Dunaliella tertiolecta Culture
Source: PLoS One. 2013 Sep 5;8(9):e72415. doi: 10.1371/journal.pone.0072415 (PMC3764108; doi:10.1371/journal.pone.0072415)
Supplement: Table S1 — The normalized ion intensities of the identified lipids over the culture conditions and time course in D. tertiolecta. (DOCX) [file pone.0072415.s009.docx]

**Table S1.** The normalized ion intensities of the identified lipids over the culture conditions and time course in *D. tertiolecta*.^a^

|  |  |  |  | CultureConditions^b^ | |  |  |  |  |  |  |  |  |  |  |  |  |  |  |  |  |  |
| --- | --- | --- | --- | --- | --- | --- | --- | --- | --- | --- | --- | --- | --- | --- | --- | --- | --- | --- | --- | --- | --- | --- |
|  |  |  |  | LLNS |  |  |  |  | LLND |  |  |  |  | HLNS |  |  |  |  | HLND |  |  |  |
|  |  |  |  | 0h | 12h | 36h | 72h |  | 0h | 12h | 36h | 72h |  | 0h | 12h | 36h | 72h |  | 0h | 12h | 36h | 72h |
| No. | Compounds^c^ | Proposed Composition | Ion Mode ^d^ |  |  |  |  |  |  |  |  |  |  |  |  |  |  |  |  |  |  |  |
| 1 | DGTS 30:2 | C 16:0/14:2 | (+) | 7.13±0.46 | 3.93±1.45 | 6.48±1.47 | 4.20±1.44 |  | 5.93±6.36 | 6.4±1.79 | 5.56±0.63 | 5.44±1.64 |  | 5.87±1.22 | 5.86±2.23 | 4.74±1.93 | 4.35±0.18 |  | 5.06±1.90 | 5.37±0.65 | 6.47±2.90 | 7.03±2.87 |
| 2 | DGTS 32:4 | C 16:0/16:4 | (+) | 2.76±0.46 | 2.63±0.36 | 3.28±0.49 | 4.96±0.18 |  | 3.99±1.96 | 4.98±0.88 | 3.60±0.11 | 5.75±2.09 |  | 3.07±0.77 | 2.77±0.12 | 2.74±0.33 | 4.17±0.56 |  | 4.17±0.92 | 4.22±1.58 | 1.84±0.06 | 3.56±0.43 |
| 3 | DGTS 32:2 | C 16:0/16:2 | (+) | 2.54±0.06 | 2.64±0.35 | 2.33±0.04 | 2.68±0.19 |  | 2.91±0.78 | 3.59±0.57 | 3.01±0.01 | 3.70±0.89 |  | 2.76±0.11 | 2.83±0.45 | 2.70±0.01 | 2.92±0.61 |  | 3.06±0.09 | 3.05±0.48 | 2.28±0.38 | 3.54±0.12 |
| 4 | DGTS 34:4 | C 18:4/16:0 | (+) | 2.06±0.22 | 2.51±0.32 | 4.24±0.19 | 6.41±0.06 |  | 3.40±2.36 | 3.58±0.34 | 2.05±0.06 | 3.92±1.76 |  | 2.72±0.95 | 3.27±0.04 | 3.35±0.34 | 5.19±0.34 |  | 4.11±1.92 | 4.22±2.29 | 1.35±0.13 | 3.30±0.66 |
| 5 | DGTS 34:3 | C 18:3/16:0 | (+) | 37.64±3.36 | 42.29±13.69 | 58.6±3.37 | 86.76±3.07 |  | 38.52±42.58 | 80.20±17.77 | 56.77±0.21 | 102.64±37.92 |  | 48.91±11.76 | 66.56±3.98 | 64.28±5.59 | 95.99±15.22 |  | 60.87±13.15 | 69.25±9.08 | 43.34±4.16 | 105.43±19.93 |
| 6 | DGTS 34:2 | C 18:2/16:0 | (+) | 22.1±2.74 | 16.95±6.53 | 20.34±1.65 | 30.17±0.23 |  | 20.33±22.62 | 43.56±13.01 | 42.54±3.93 | 54.02±11.47 |  | 25.31±3.35 | 29.56±1.92 | 27.5±0.51 | 40.84±7.22 |  | 30.91±4.15 | 42.07±4.89 | 40.01±2.30 | 80.91±14.02 |
| 7 | DGTS 34:1 | C 18:1/16:0 | (+) | 6.01±0.10 | 4.14±1.05 | 5.00±0.03 | 7.27±0.30 |  | 5.38±4.99 | 10.50±2.74 | 10.10±1.53 | 10.56±2.00 |  | 6.55±0.90 | 6.79±0.63 | 6.39±0.05 | 8.09±1.01 |  | 7.86±1.18 | 9.59±1.46 | 9.52±0.31 | 15.53±2.07 |
| 8 | DGTS 36:6 | C 18:3/18:3 | (+) | 5.79±1.37 | 5.69±1.3 | 12.04±1.96 | 19.68±0.62 |  | 8.54±7.59 | 9.41±2.33 | 7.00±1.19 | 17.59±6.90 |  | 7.60±2.09 | 6.12±0.50 | 6.77±0.84 | 13.48±1.72 |  | 10.52±3.81 | 9.48±5.10 | 3.34±0.10 | 13.99±1.93 |
| 9 | DGTS 36:5 | C 18:2/18:3 | (+) | 7.23±0.97 | 7.34±1.31 | 13.93±0.65 | 17.46±0.60 |  | 10.98±11.46 | 14.64±4.96 | 10.85±1.55 | 22.25±6.43 |  | 9.36±2.85 | 11.91±1.12 | 11.50±1.03 | 15.36±2.36 |  | 14.30±5.81 | 14.88±7.25 | 6.01±0.43 | 23.17±2.50 |
| 10 | DGTS 36:4 | C 18:2/18:2 | (+) | 5.19±0.50 | 5.00±0.48 | 8.13±0.08 | 10.43±0.50 |  | 7.11±6.64 | 10.86±3.91 | 7.78±0.93 | 12.75±2.62 |  | 6.52±1.94 | 9.61±0.82 | 8.48±0.37 | 9.02±0.99 |  | 9.19±3.04 | 9.96±3.13 | 5.15±0.68 | 16.10±1.46 |
| 11 | DGTS 36:3 | C 18:1/18:2 | (+) | 10.58±2.28 | 12.04±2.51 | 13.45±0.62 | 16.16±3.73 |  | 9.38±0.73 | 14.63±1.60 | 11.69±2.80 | 16.50±2.90 |  | 8.12±2.75 | 10.98±2.24 | 9.29±0.14 | 9.86±0.93 |  | 12.83±2.76 | 11.67±0.34 | 7.01±0.04 | 14.54±1.38 |
| 12 | DGTS 36:2 | C18:1/18:1, C18:2/18:0 | (+) | 6.65±1.09 | 9.14±3.56 | 7.30±0.07 | 8.79±2.02 |  | 4.06±0.70 | 7.69±0.18 | 7.15±1.20 | 9.25±0.71 |  | 5.13±1.02 | 7.69±1.56 | 6.92±0.58 | 6.65±0.45 |  | 6.84±0.65 | 6.71±0.76 | 5.56±0.29 | 8.40±0.11 |
| 13 | LysoDGTS 16:0 | C 16:0 | (+) | 83.71±1.21 | 70.42±25.59 | 71.87±17.53 | 34.8±6.11 |  | 148.16±75.89 | 71.81±2.80 | 74.54±5.26 | 30.45±3.99 |  | 74.56±0.38 | 76.03±0.64 | 87.53±3.76 | 34.65±2.27 |  | 94.05±19.47 | 50.36±3.93 | 60.93±5.70 | 29.28±7.63 |
| 14 | LysoDGTS 18:3 | C 18:3 | (+) | 23.49±0.53 | 19.03±7.26 | 25.15±3.79 | 26.97±4.25 |  | 50.97±28.03 | 20.06±0.88 | 19.67±0.10 | 16.85±2.42 |  | 23.21±0.33 | 20.38±0.59 | 24.43±1.91 | 19.53±0.14 |  | 29.63±7.15 | 18.45±4.55 | 16.68±0.63 | 12.70±2.26 |
| 15 | MGDG 34:7 | C 18:3/16:4 | (+) | 5.42±1.81 | 7.82±2.24 | 2.73±0.73 | 5.07±0.78 |  | 4.35±1.91 | 3.09±0.06 | 3.30±0.70 | 4.08±1.16 |  | 4.27±0.01 | 2.83±1.09 | 2.73±0.59 | 2.73±0.48 |  | 5.46±0.58 | 3.46±0.06 | 2.55±0.17 | 2.81±0.09 |
|  |  |  | (-) | 2.33±0.68 | 3.30±0.38 | 3.26±1.32 | 2.67±0.14 |  | 1.98±0.06 | 2.78±0.24 | 2.93±0.24 | 2.71±0.39 |  | 2.01±0.19 | 3.12±0.40 | 1.94±0.18 | 2.34±0.32 |  | 2.32±0.53 | 2.69±0.87 | 2.41±0.50 | 2.77±0.29 |
| 16 | MGDG 34:6 | C 18:3/16:3 | (-) | 0.84±0.26 | 1.31±0.35 | 1.12±0.43 | 0.92±0.03 |  | 0.70±0.01 | 0.93±0.10 | 1.07±0.13 | 0.99±0.18 |  | 0.74±0.00 | 1.38±0.14 | 0.94±0.14 | 1.00±0.17 |  | 1.14±0.63 | 1.31±0.82 | 1.00±0.22 | 1.19±0.12 |
| 17 | DGDG 34:7 | C 18:3/16:4 | (+) | 5.37±0.03 | 5.77±1.52 | 3.10±0.20 | 3.67±0.25 |  | 3.55±1.46 | 4.40±0.26 | 5.36±0.55 | 6.13±0.32 |  | 4.24±0.36 | 4.88±1.32 | 4.71±1.47 | 4.61±0.27 |  | 4.90±0.56 | 4.36±0.81 | 5.79±0.07 | 5.85±0.53 |
| 18 | DGDG 34:6 | C 18:3/16:3 | (+) | 7.17±0.64 | 7.57±2.13 | 3.45±0.13 | 3.84±0.19 |  | 4.19±1.74 | 4.92±0.49 | 6.02±1.17 | 6.63±0.09 |  | 5.22±0.10 | 5.90±1.95 | 5.32±1.75 | 5.20±0.30 |  | 6.28±0.96 | 5.11±1.03 | 7.13±0.75 | 7.45±0.12 |
| 19 | DGDG 34:5 | C 18:3/16:2 | (+) | 5.21±0.24 | 5.78±1.96 | 2.66±0.17 | 2.98±0.10 |  | 2.66±0.52 | 4.03±0.30 | 5.72±1.36 | 5.49±0.14 |  | 3.71±0.16 | 4.39±1.49 | 3.95±1.31 | 4.26±0.08 |  | 4.67±0.47 | 5.01±0.87 | 7.89±0.67 | 7.30±0.08 |
| 20 | DGDG 34:4 | C18:4/16:0, C18:3/16:1, C18:2.16:2, C18:1/16:3 | (+) | 5.42±0.34 | 5.54±1.34 | 2.65±0.08 | 2.92±0.03 |  | 2.81±0.43 | 3.86±0.27 | 5.36±1.18 | 5.02±0.20 |  | 4.06±0.13 | 4.01±1.24 | 3.58±1.20 | 3.81±0.01 |  | 4.99±0.52 | 4.88±0.54 | 7.04±0.39 | 6.27±0.01 |
| 21 | DGDG 34:3 | C 18:3/16:0 | (+) | 6.07±0.44 | 7.57±1.58 | 5.10±0.41 | 5.31±0.29 |  | 3.02±0.44 | 4.97±0.03 | 5.62±0.95 | 5.33±0.24 |  | 5.38±0.05 | 4.59±1.12 | 4.15±1.02 | 4.20±0.08 |  | 6.25±0.81 | 5.12±0.77 | 5.58±0.13 | 4.99±0.08 |
| 22 | DGDG 34:2 | C 18:2/16:0 | (+) | 3.79±0.02 | 5.79±1.54 | 4.67±0.04 | 5.26±0.68 |  | 2.16±1.13 | 3.96±0.04 | 3.72±0.41 | 3.73±0.38 |  | 4.21±0.15 | 3.24±0.62 | 3.00±0.55 | 3.64±1.16 |  | 4.84±0.16 | 3.71±0.51 | 2.86±0.05 | 2.51±0.10 |
| 23 | SQDG 32:0 | C 16:0/16:0 | (-) | 0.66±0.20 | 1.07±0.57 | 1.12±0.06 | 1.04±0.35 |  | 0.62±0.03 | 0.85±0.02 | 0.98±0.08 | 0.93±0.13 |  | 0.63±0.05 | 1.35±0.16 | 1.23±0.15 | 1.41±0.20 |  | 0.81±0.20 | 1.11±0.19 | 1.49±0.11 | 1.54±0.06 |
| 24 | SQDG 34:3 | C 18:3/16:0 | (-) | 2.38±0.78 | 3.74±1.72 | 4.46±0.56 | 3.74±0.99 |  | 2.46±0.01 | 3.32±0.18 | 3.33±0.25 | 2.60±0.28 |  | 2.29±0.19 | 4.56±0.37 | 3.87±0.53 | 3.63±0.43 |  | 3.13±0.66 | 3.16±0.27 | 3.31±0.46 | 2.57±0.01 |
| 25 | SQDG 34:2 | C 18:2/16:0 | (-) | 1.19±0.47 | 2.19±1.32 | 2.18±0.34 | 1.67±0.41 |  | 1.20±0.02 | 1.61±0.03 | 1.81±0.27 | 1.40±0.01 |  | 1.17±0.06 | 2.85±0.12 | 2.40±0.55 | 1.80±0.33 |  | 2.09±1.09 | 2.27±1.04 | 2.05±0.42 | 1.54±0.02 |
| 26 | SQDG 34:1 | C 18:1/16:0 | (-) | 0.61±0.17 | 0.98±0.67 | 0.68±0.08 | 0.57±0.10 |  | 0.54±0.00 | 0.70±0.03 | 0.75±0.09 | 0.55±0.01 |  | 0.58±0.01 | 1.36±0.06 | 1.11±0.38 | 0.82±0.06 |  | 0.92±0.37 | 1.05±0.36 | 1.07±0.29 | 0.76±0.06 |
| 27 | PI 34:1 | C 18:1/16:0 | (-) | 0.34±0.14 | 0.55±0.30 | 0.34±0.05 | 0.32±0.03 |  | 0.25±0.01 | 0.29±0.01 | 0.43±0.04 | 0.35±0.02 |  | 0.28±0.01 | 0.57±0.05 | 0.42±0.09 | 0.40±0.01 |  | 0.31±0.10 | 0.49±0.23 | 0.55±0.10 | 0.50±0.00 |
| 28 | PG 34:3 | C 18:3/16:0 | (-) | 0.45±0.09 | 0.76±0.27 | 1.02±0.20 | 1.03±0.20 |  | 0.46±0.01 | 0.51±0.01 | 0.55±0.01 | 0.61±0.00 |  | 0.44±0.03 | 0.81±0.08 | 0.58±0.09 | 0.65±0.06 |  | 0.60±0.08 | 0.42±0.06 | 0.49±0.14 | 0.64±0.09 |
| 29 | PG 34:2 | C 18:1/16:1 | (-) | 0.37±0.09 | 0.75±0.42 | 0.67±0.15 | 0.71±0.16 |  | 0.37±0.02 | 0.50±0.01 | 0.60±0.04 | 0.62±0.03 |  | 0.35±0.01 | 0.87±0.08 | 0.62±0.13 | 0.67±0.14 |  | 0.53±0.18 | 0.51±0.14 | 0.57±0.13 | 0.68±0.08 |

^a^Each value represents the mean ± standard deviation (SD) (n=2).

^b^LLNS, low light intensity and nitrate sufficiency; LLND, low light intensity and nitrate deficiency; HLNS, high light intensity and nitrate sufficiency; HLND, high light intensity and nitrate deficiency.

^c^DGTS, diacylglyceryltrimethylhomoserine; MGDG, monogalactosyldiacylglycerol; DGDG, digalactosyldiacylglycerol; SQDG, sulfoquinovosyldiacylglycerol; PI, phosphatidylinositol; PG, phosphatidylglycerol.

^d^For simplicity, ‘x10e5’ and ‘x10e3’ were omitted from the values of identified lipids in positive mode and negative mode, respectively.
