# Supplementary material for: Effects of Light Intensity and Nitrogen Starvation on Glycerolipid, Glycerophospholipid, and Carotenoid Composition in Dunaliella tertiolecta Culture
Source: PLoS One. 2013 Sep 5;8(9):e72415. doi: 10.1371/journal.pone.0072415 (PMC3764108; doi:10.1371/journal.pone.0072415)
Supplement: Table S2 — Regression equations, correlation coefficients (r2 values), LOD, and LOQ of carotenoids. (DOCX) [file pone.0072415.s010.docx]

**Table S2.** Regression equations, correlation coefficients (r^2^ values), LOD, and LOQ of carotenoids.

| Compound^a^ | Regression equation | r^2^ values | LOD^b^ (μg/mL) | LOQ^c^(μg/mL) |
| --- | --- | --- | --- | --- |
| NEO | y=0.0626x-0.0005 | 0.9999 | 0.105 | 0.319 |
| VIO | y=0.1728x-0.0011 | 0.9997 | 0.020 | 0.061 |
| ANT | y=0.1123x-0.1364 | 0.9959 | 0.101 | 0.306 |
| LUT | y=0.0865x-0.0097 | 0.9996 | 0.167 | 0.507 |
| γCAR | y=0.1971x+0.0040 | 0.9995 | 0.164 | 0.496 |
| αCAR | y=0.3474x-0.0289 | 0.9993 | 0.063 | 0.190 |
| βCAR | y=0.3085x-0.0717 | 0.9993 | 0.039 | 0.117 |

^a^NEO, neoxanthin; VIO, violaxanthin; ANT, anthraxanthin; LUT, lutein; γCAR, γ-carotene; αCAR, α-carotene; βCAR, β-carotene.

^b^LOD, limit of detection.

^c^LOQ, limit of quantitation.
